# Alpha-synuclein is a DNA binding protein that modulates DNA repair with implications for Lewy body disorders

**DOI:** 10.1038/s41598-019-47227-z

**Published:** 2019-07-29

**Authors:** Allison J. Schaser, Valerie R. Osterberg, Sydney E. Dent, Teresa L. Stackhouse, Colin M. Wakeham, Sydney W. Boutros, Leah J. Weston, Nichole Owen, Tamily A. Weissman, Esteban Luna, Jacob Raber, Kelvin C. Luk, Amanda K. McCullough, Randall L. Woltjer, Vivek K. Unni

**Affiliations:** 1 Department of Neurology & Jungers Center for Neurosciences Research, Oregon Health & Science University, Portland, OR 97239 USA; 2 Neuroscience Graduate Program, Vollum Institute, Oregon Health & Science University, Portland, OR 97239 USA; 3 Departments of Behavioral Neuroscience, Neurology, and Radiation Medicine and Division of Neuroscience, ONPRC, Oregon Health & Science University, Portland, Oregon 97239 USA; 4 Department of Molecular and Medical Genetics, Oregon Health & Science University, Portland, OR 97239 USA; 5 Department of Biology, Lewis & Clark College, Portland, OR 97219 USA; 6 Department of Pathology and Laboratory Medicine and Center for Neurodegenerative Disease Research, University of Pennsylvania Perelman School of Medicine, Philadelphia, PA 19104 USA; 7 Oregon Institute of Occupational Health Sciences, Oregon Health & Science University, Portland, OR 97239 USA; 8 Department of Pathology, Division of Neuropathology, Oregon Health & Science University, Portland, OR 97239 USA; 9 OHSU Parkinson Center, Oregon Health & Science University, Portland, OR 97239 USA

**Keywords:** Dementia, Parkinson's disease

## Abstract

Alpha-synuclein is a presynaptic protein that forms abnormal cytoplasmic aggregates in Lewy body disorders. Although nuclear alpha-synuclein localization has been described, its function in the nucleus is not well understood. We demonstrate that alpha-synuclein modulates DNA repair. First, alpha-synuclein colocalizes with DNA damage response components within discrete foci in human cells and mouse brain. Removal of alpha-synuclein in human cells leads to increased DNA double-strand break (DSB) levels after bleomycin treatment and a reduced ability to repair these DSBs. Similarly, alpha-synuclein knock-out mice show increased neuronal DSBs that can be rescued by transgenic reintroduction of human alpha-synuclein. Alpha-synuclein binds double-stranded DNA and helps to facilitate the non-homologous end-joining reaction. Using a new, *in vivo* imaging approach that we developed, we find that serine-129-phosphorylated alpha-synuclein is rapidly recruited to DNA damage sites in living mouse cortex. We find that Lewy inclusion-containing neurons in both mouse model and human-derived patient tissue demonstrate increased DSB levels. Based on these data, we propose a model whereby cytoplasmic aggregation of alpha-synuclein reduces its nuclear levels, increases DSBs, and may contribute to programmed cell death via nuclear loss-of-function. This model could inform development of new treatments for Lewy body disorders by targeting alpha-synuclein-mediated DNA repair mechanisms.

## Introduction

Alpha-synuclein is a small, 140-amino acid protein that localizes to presynaptic terminals, where it regulates neurotransmitter vesicle cycling. In addition to having specific functions in modulating vesicle exocytosis^1^ and endocytosis^2^, under pathological conditions alpha-synuclein aggregates into somatic and neuritic inclusions. This aggregation occurs in neurons in Parkinson’s Disease (PD) and related Lewy body disorders (e.g. Dementia with Lewy Bodies), and in oligodendrocytes in Multiple System Atrophy. It is currently unclear how cytoplasmic aggregation of alpha-synuclein into Lewy body pathology is related to the neuronal death that characterizes these neurodegenerative diseases. Since its discovery, alpha-synuclein has been argued to also be present in the nucleus of neurons^3,4^, which could suggest that it plays additional cellular roles beyond the synapse. This topic has been controversial, however, since early reports were^5–7^ or were not^8,9^ able to confirm alpha-synuclein’s nuclear localization. The cause of this discrepancy was likely due, in part, to the fact that some antibodies used to detect alpha-synuclein in the nucleus by immunohistochemistry can cross-react with nuclear epitopes that are not alpha-synuclein^10^. More recent studies using chromatin immunoprecipitation^11^, subcellular fractionation^12,13^, or fluorescently-tagged proteins^14^, have shown that alpha-synuclein can be found in the nucleus and that its presence there is regulated by several factors, including oxidative stress^11,12^ and post-translational modifications of the protein^13,15^.

In contrast to its presynaptic actions modulating neurotransmitter vesicle recycling, the potential function of nuclear alpha-synuclein is not as well understood. However, many of the proposed activities of nuclear alpha-synuclein involve either direct or indirect interactions with DNA, including modulating histone modification state^5,16^ or direct DNA binding^17–20^. Some forms of aggregated alpha-synuclein have been shown to have DNA endonuclease activity^18^. In overexpression models, which increase specific aggregated forms of alpha-synuclein potentially relevant to disease, neuronal toxicity is increased, possibly due to downregulated transcription of DNA repair genes^21^, or increases in pro-oxidant species that result in DNA damage^22^. Nuclear alpha-synuclein has also been shown to influence neuronal cell death; in *Drosophila*, nuclear forms of the protein promoted neurotoxicity^16,23^, while in human cells higher molecular weight alpha-synuclein species in the nucleus were associated with reduced toxicity^13^. Alpha-synuclein’s interaction with DNA has also been argued to regulate normal cell function by influencing transcription^11,19,24^. Despite this important previous work, we still lack a clear picture of why alpha-synuclein is present in the nucleus of many neural and non-neural cell types, and how its nuclear function/s might be disrupted in neurodegenerative diseases where cytoplasmic alpha-synuclein aggregation occurs.

Our previous work using *in vivo* multiphoton imaging in a mouse model of parkinsonism demonstrated that cortical Lewy inclusion formation coincided with the loss of soluble alpha-synuclein from both the cytoplasm and nucleus of inclusion-bearing neurons^25^. This suggests that cytoplasmic alpha-synuclein aggregation may decrease the amount of protein available for any nuclear or cytoplasmic role it may play, contributing to a loss-of-function. Interestingly, there are potential functional parallels between alpha-synuclein and a known DNA repair protein PC4 (yeast ortholog SUB1), which is involved in coordinating cellular responses to DNA double-strand breaks (DSBs). Similar to alpha-synuclein, PC4 is a small, 127-amino acid protein, with intrinsically unstructured domains^26^, DNA binding and transcriptional modulatory activities^27^. In addition, PC4/SUB1 functions as a regulator of cellular responses to oxidative stress, with the ability to protect DNA from oxidative damage^28^ and to coordinate specific forms of DSB repair^29^. While alpha-synuclein and PC4 share several basic features, to the best of our knowledge, functions in DNA repair have not been reported for alpha-synuclein. In addition, given the data showing that alpha-synuclein can directly bind DNA^17–20^; PD is linked to DNA damaging insults like oxidative stress^30^; and oxidative stress increases nuclear alpha-synuclein levels^31^, we set out to test whether alpha-synuclein plays a role in regulating normal cellular responses to DNA damage and whether this function could be compromised in Lewy inclusion-bearing cells.

We therefore assessed whether alpha-synuclein could be involved in the DNA damage response (DDR) pathway. We demonstrate here that alpha-synuclein plays an unexpected normal function in the nucleus in regulating DNA repair, including DSB repair, and that this function may be compromised in Lewy inclusion-bearing neurons, therefore playing a potential role in triggering cell death. This mechanism provides a new link between alpha-synuclein-rich Lewy body formation and neurodegeneration.

## Results

### Alpha-synuclein forms discrete nuclear foci that colocalize with known DDR components

**Figure 1:**
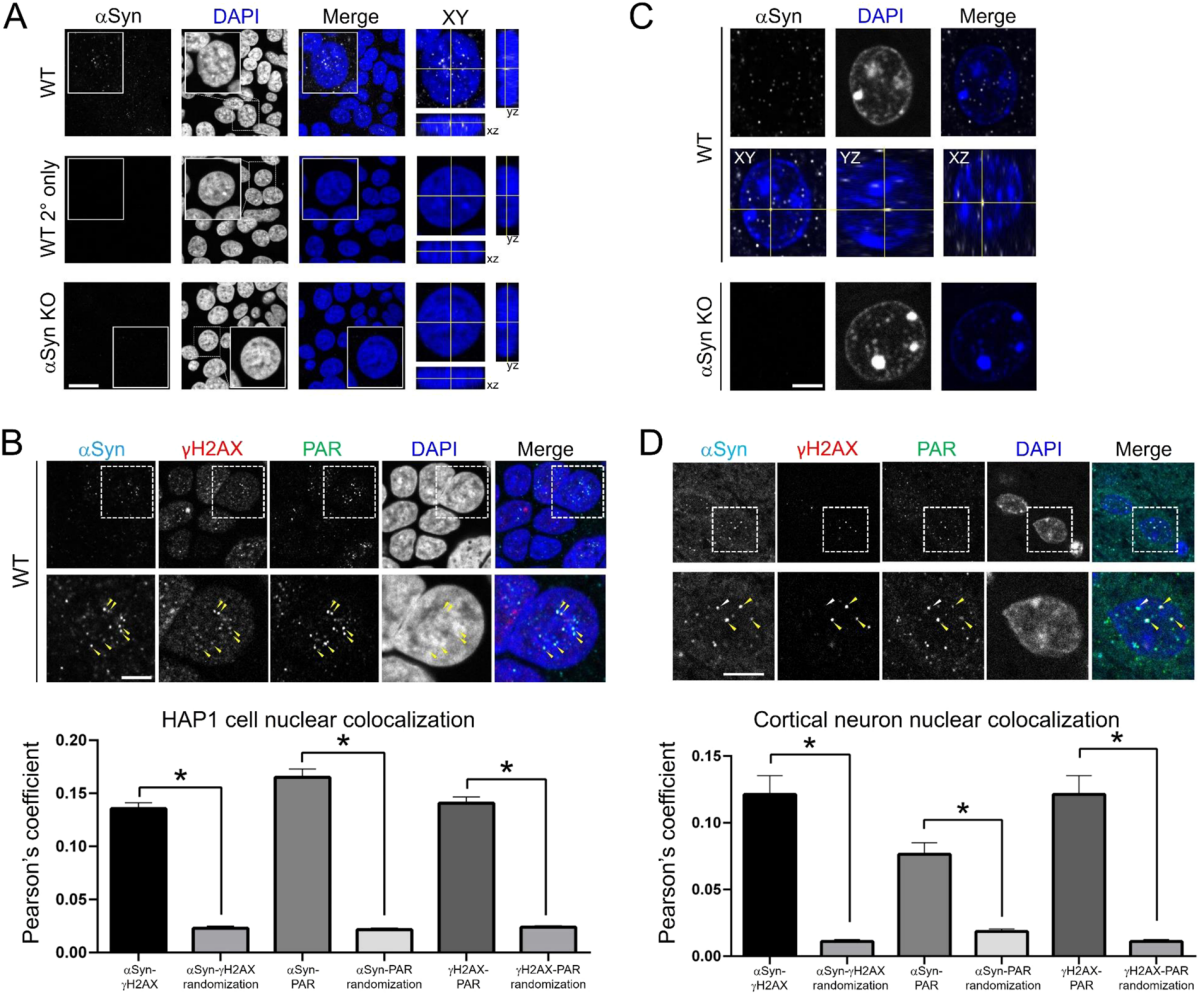
Alpha-synuclein forms discrete nuclear foci that colocalize with known DDR components. (**A**) Top: A representative image shows that endogenous alpha-synuclein (αSyn) forms discrete foci in HAP1 cell nuclei that are localized within the nucleus. Scale bar 20 μm, inset 10 μm. Middle & Bottom: No such similar staining is seen with a secondary antibody-only control or in SNCA knock-out (αSyn KO) cells. (**B**) Top: A representative image shows that intranuclear αSyn foci colocalize with DDR components, including the DNA repair factors γH2AX and PAR. Inset shows region shown at higher magnification below. Scale bar 5 μm. Bottom: Quantification of colocalization between αSyn, γH2AX and PAR compared to what would be expected with the same foci density at random (αSyn-γH2AX = 0.136 ± 0.005, random translation = 0.023 ± 0.002, N = 322 nuclei, paired t-test p < 0.0001; αSyn-PAR = 0.165 ± 0.008, random translation = 0.022 ± 0.001, N = 325 nuclei, paired t-test p < 0.0001; γH2AX-PAR = 0.141 ± 0.006, random translation = 0.024 ± 0.001, N = 325 nuclei, paired t-test p < 0.0001). (**C**) Endogenous mouse αSyn staining in WT animals is specific and can be found as discrete foci within the nucleus of cortical neurons. Top: Images demonstrate a single plane from a three dimensional z-stack showing αSyn foci. Middle: Projection of three dimensional z-stack in 3 different planes shows that αSyn foci are within the nucleus. Bottom: αSyn staining is absent in SNCA KO mouse tissue, since it is completely abolished in all (>200) αSyn KO mouse cortical neurons analyzed, as shown in this representative image. Scale bar 4 μm. (**D**) Top: A representative image shows that endogenous mouse αSyn forms discrete intranuclear foci in mouse cortical neurons that colocalize with DDR components, including the DNA repair factors γH2AX and PAR. Inset shows region shown at higher magnification below. Scale bar 5 μm. Bottom: Quantification of colocalization between αSyn, γH2AX and PAR compared to what would be expected with the same foci density at random (αSyn-γH2AX = 0.121 ± 014, random translation = 0.011 ± 0.002, paired t-test p < 0.0001; αSyn-PAR = 0.076 ± 0.002, random translation = 0.019 ×  ± 0.002, paired t-test p < 0.0001; γH2AX-PAR = 0.121 ± 0.014, random translation = 0.011 ± 0.002, paired t-test p < 0.0001; N = 79 nuclei, 4 animals).

To begin to assess whether alpha-synuclein plays a role in DNA repair, we used immunocytochemistry (ICC) to test whether it localizes to sites of DNA damage within the nucleus of human HAP1 cells. The widely used antibody Syn1 revealed that alpha-synuclein was present within multiple, small discrete foci within the nucleus (Fig. 1A). We used this antibody after testing a panel of five antibodies, because Syn1 provided clear nuclear staining that was absent in alpha-synuclein gene (SNCA) knock-out cells, confirming its specificity (Fig. 1A Supplemental). Given the presence of alpha-synuclein nuclear foci and the intriguing potential similarities between alpha-synuclein and PC4/SUB1, a protein known to be involved in coordinating DSB repair^29^, we next tested for colocalization between nuclear alpha-synuclein and established markers of DNA damage. We found that nuclear alpha-synuclein foci colocalized with both the DSB repair factor phosphorylated histone 2 A.X (γH2AX); and a factor involved in both single- and double-strand break repair, poly-ADP ribose (PAR) polymer (Fig. 1B). Notably, the signals from all three often triple-localized within individual nuclear foci (Fig. 1B).

We next wished to test whether alpha-synuclein would also colocalize with these DNA damage markers in cortical neurons in fixed tissue sections. Again using the specific Syn1 antibody (following another analysis of available antibodies in mouse cortex; Table 1 Supplemental), we detected multiple, discrete alpha-synuclein foci within neuronal nuclei in wild-type (WT) mice (Fig. 1C), with staining very similar to the pattern seen in HAP1 cells. We stained WT mouse cortical sections with the neuronal marker NeuN to test whether the alpha-synuclein nuclear foci were present in neurons and/or glial cells. This showed that nuclear alpha-synuclein foci are present in neurons in the mouse cortex and are relatively absent from glial cells (Fig. 1B Supplemental). We next tested whether nuclear alpha-synuclein foci in mouse cortical neurons colocalized with DNA damage factors, as we detected in HAP1 cells (Fig. 1B). As in HAP1 cells, alpha-synuclein nuclear foci significantly colocalized with foci composed of γH2AX and PAR in WT mice (Fig. 1D). Taken together, these data strongly suggest that alpha-synuclein forms discrete, nuclear foci that colocalize with known markers of DNA breaks in both mouse cortical neurons and human HAP1 cells, raising the possibility that alpha-synuclein is localizing to damaged regions of DNA.

### Alpha-synuclein knock-out in HAP1 cells compromises DSB repair

**Figure 2:**
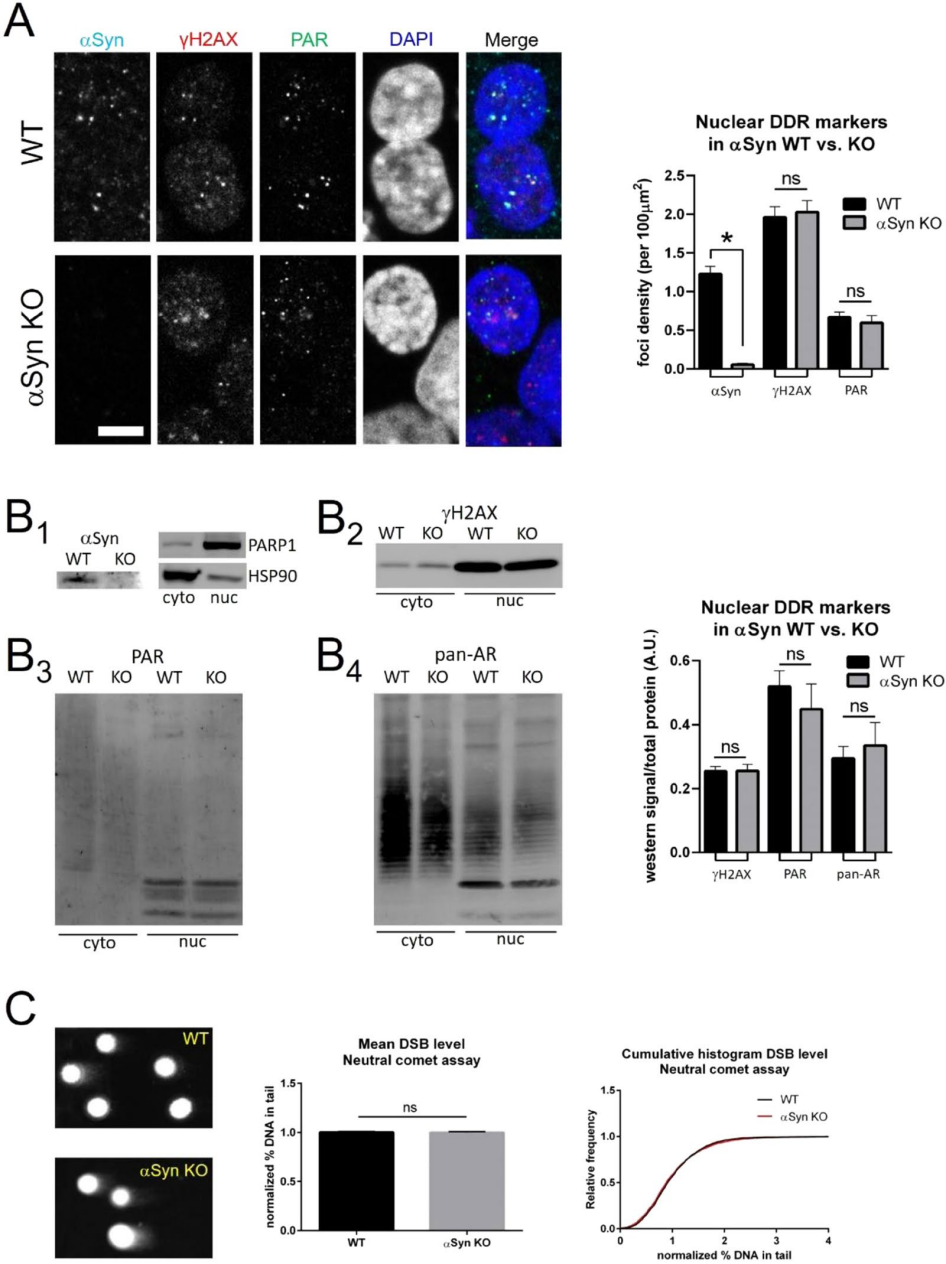
Alpha-synuclein knock-out does not alter DSB levels at baseline in HAP1 cells. (**A**) SNCA knock-out (αSyn KO) does not alter baseline levels of nuclear γH2AX or PAR foci (foci density per 100 μm^2^): WT αSyn = 1.23 ± 0.10, αSyn KO αSyn = 0.06 ± 0.02, unpaired t-test p < 0.0001; WT γH2AX = 1.96 ± 0.14, αSyn KO αSyn = 2.03 ± 0.15, unpaired t-test p = 0.7422; WT PAR = 0.67 ± 0.07, αSyn KO PAR = 0.60 ± 0.09, unpaired t-test p = 0.5452; WT N = 326 nuclei, αSyn KO N = 252 nuclei). Scale bar 5 μm. (**B**) Left: (B_1_) Western blotting shows expected absence of αSyn protein in αSyn KO cells. Subcellular fractionation was used to purify nuclear and cytoplasmic proteins, as demonstrated by relative enrichment of the nuclear protein PARP1 in the nuclear fraction and the cytosolic protein HSP90 in the cytoplasmic fraction. Using this approach, blotting for nuclear γH2AX (B_2_), PAR (B_3_) and pan-(mono- & poly-) ADP-ribose (B_4_) showed no significant difference between WT and αSyn KO cells at baseline, when normalized to total protein levels (using REVERT stain, not shown). Right: Group data: WT γH2AX = 0.25 ± 0.01, αSyn KO γH2AX = 0.26 ± 0.02, unpaired t-test p = 0.9682; WT PAR = 0.52 ± 0.05, αSyn KO PAR = 0.45 ± 0.08, unpaired t-test p = 0.4883; WT pan-AR = 0.29 ± 0.04, αSyn KO pan-AR = 0.34 ± 0.07, unpaired t-test p = 0.6365; WT N = 3, αSyn KO N = 3 biological replicates). (**C**) Neutral comet assay shows no difference in levels of DSBs between WT and αSyn KO cells at baseline. Left: Comet images, middle: group data (normalized % DNA in tail: WT = 1.00 ± 0.01, αSyn KO = 1.00 ± 0.01, unpaired t-test p = 0.8860; WT N = 3016, αSyn KO N = 4171), right: cumulative probability histogram showing superimposable distributions.

**Figure 3:**
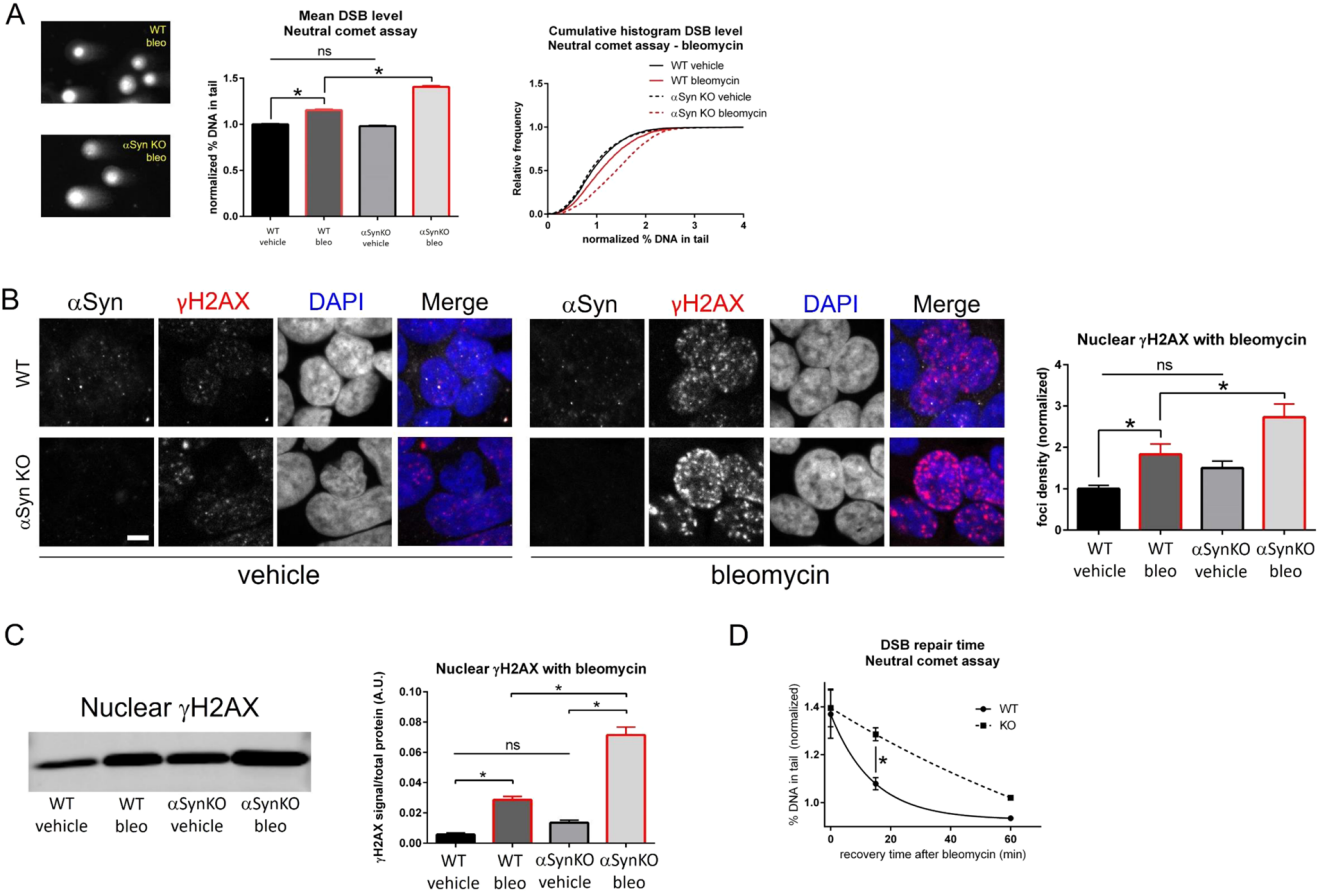
Alpha-synuclein knock-out in HAP1 cells compromises DSB repair after bleomycin treatment. (**A**) Neutral comet assay after treatment with bleomycin (10μg/mL for 60 min) shows greater levels of DSBs in SNCA KO (αSyn KO) cells compared to WT. Left: Comet images, middle: group data (normalized % DNA in tail: WT vehicle = 1.00 ± 0.01 N = 4561 nuclei, WT bleo = 1.15 ± 0.01 N = 2269 nuclei, αSyn KO vehicle = 0.98 ± 0.01 N = 5182 nuclei, αSyn KO bleo = 1.41 ± 0.01 N = 2734 nuclei; F(3, 14742) = 394.83, p < 0.0001, ANOVA, post-hoc Tukey test: WT vehicle vs. WT bleo p < 0.0001, WT vehicle vs. αSyn KO vehicle p = 0.3487, WT vehicle vs. αSyn KO bleo p < 0.0001, WT bleo vs. αSyn KO vehicle p < 0.0001, WT bleo vs. αSyn KO bleo p < 0.0001, αSyn KO vehicle vs. αSyn KO bleo p < 0.0001, several significance lines have been left off the figure to improve clarity). Red outline on bars represents bleomycin treated conditions. Right: cumulative probability histogram showing differences in the distributions. (**B**) Bleomycin treatment (10μg/mL for 60 min) induces greater DSB levels in αSyn KO cells compared to WT, as measured by normalized nuclear γH2AX levels (normalized foci density): WT vehicle = 1.00 ± 0.08 N = 215 nuclei, WT bleo = 1.83 ± 0.25 N = 117 nuclei, αSyn KO vehicle = 1.51 ± 0.16 N = 159 nuclei, αSyn KO bleo = 2.73 ± 0.32 N = 117 nuclei; F(3, 604) = 14.564, p < 0.0001, ANOVA, post-hoc Tukey test: WT vehicle vs. WT bleo p = 0.0103, WT vehicle vs. αSyn KO vehicle p = 0.1570, WT vehicle vs. αSyn KO bleo p < 0.0001, WT bleo vs. αSyn KO vehicle p = 0.6621, WT bleo vs. αSyn KO bleo p = 0.0159, αSyn KO vehicle vs. αSyn KO bleo p < 0.0001. Several significance lines have been left off the figure to improve clarity. Scale bar 5 μm. Red outline on bars represents bleomycin treated conditions. (**C**) Nuclear fractionation and western blotting after treatment with bleomycin demonstrates greater nuclear γH2AX levels in αSyn KO cells compared to WT (γH2AX levels normalized to total protein levels using REVERT stain, not shown, A.U.): WT vehicle γH2AX = 0.0074 ± 0.014, WT bleo = 0.0274 ± 0.024, αSyn KO vehicle = 0.0168 ± 0.101, αSyn KO bleo = 0.0737 ± 0.036; N = 3 biological replicates, F(3, 20) = 125.52, p < 0.0001, ANOVA, post-hoc Tukey test: WT vehicle vs. WT bleo p = 0.0002, WT vehicle vs. αSyn KO vehicle p = 0.0867, WT vehicle vs. αSyn KO bleo p < 0.0001, WT bleo vs. αSyn KO vehicle p = 0.0447, WT bleo vs. αSyn KO bleo p < 0.0001, αSyn KO vehicle vs. αSyn KO bleo p < 0.0001. Several significance lines have been left off the figure to improve clarity. Red outline on bars represents bleomycin treated conditions. (**D**) Neutral comet assay analysis of recovery after removal of bleomycin shows delayed DSB repair in αSyn KO cells compared to WT (mean % DNA in tail 15 min after bleo removal: WT = 1.079 ± 0.026 N = 3 biological replicates, αSyn KO = 1.285 ± 0.027 N = 3 biological replicates; unpaired t-test p = 0.0052).

If alpha-synuclein plays a role in DSB repair, we would expect that removal of this protein would impact the DNA repair process in cells. Thus, we used a combination of approaches to test whether the DSB response was abnormal in SNCA knock-out HAP1 cells at baseline versus following induced DNA damage. At baseline, we detected no significant differences in nuclear γH2AX or PAR levels between WT and SNCA knock-out cells using ICC (Fig. 2A) and nuclear fractionation followed by western blot (Fig. 2B). In addition, direct measurement of DNA DSB levels in HAP1 WT and SNCA knock-out cells using the neutral comet assay did not detect any significant differences at baseline (Fig. 2C). In order to test whether the DDR may be compromised in SNCA knock-out cells following induced DNA damage, we treated WT and SNCA knock-out cells with the chemotherapeutic agent bleomycin (10μg/mL for 60 min), a compound known to preferentially induce DSBs in DNA. Using three separate assays, we detected increased DSBs in SNCA knock-out cells following bleomycin treatment. Neutral comet assays showed that SNCA knock-out cells had higher levels of DSBs after bleomycin treatment than WT cells (Fig. 3A). ICC analysis of nuclear DNA damage levels by γH2AX staining showed that our bleomycin treatment significantly increased γH2AX in WT cells (Fig. 3B); SNCA knock-out cells were more affected by bleomycin treatment and had an even larger γH2AX increase than WT cells (Fig. 3B). This significant increase in nuclear γH2AX levels in SNCA knock-out versus WT cells after bleomycin treatment was also seen after nuclear fractionation and western blot analysis (Fig. 3C). To test whether the increased DSB levels we measured in SNCA knock-out cells were due to a deficiency in the DSB repair process in these cells, as opposed to an increased sensitivity of the DNA to bleomycin, we treated cells with bleomycin as before (10 μg/mL for 60 min) and then removed the bleomycin, returning cells to normal medium for increasing lengths of time and measured DSB levels using the neutral comet assay. We found that bleomycin-induced DSBs were repaired more quickly in WT than SNCA knock-out cells, suggesting that the presence of alpha-synuclein facilitates DSB repair (Fig. 3D).

### Removal of alpha-synuclein in mice increases cortical neuron DSBs, which can be rescued by transgenic expression of human alpha-synuclein

**Figure 4:**
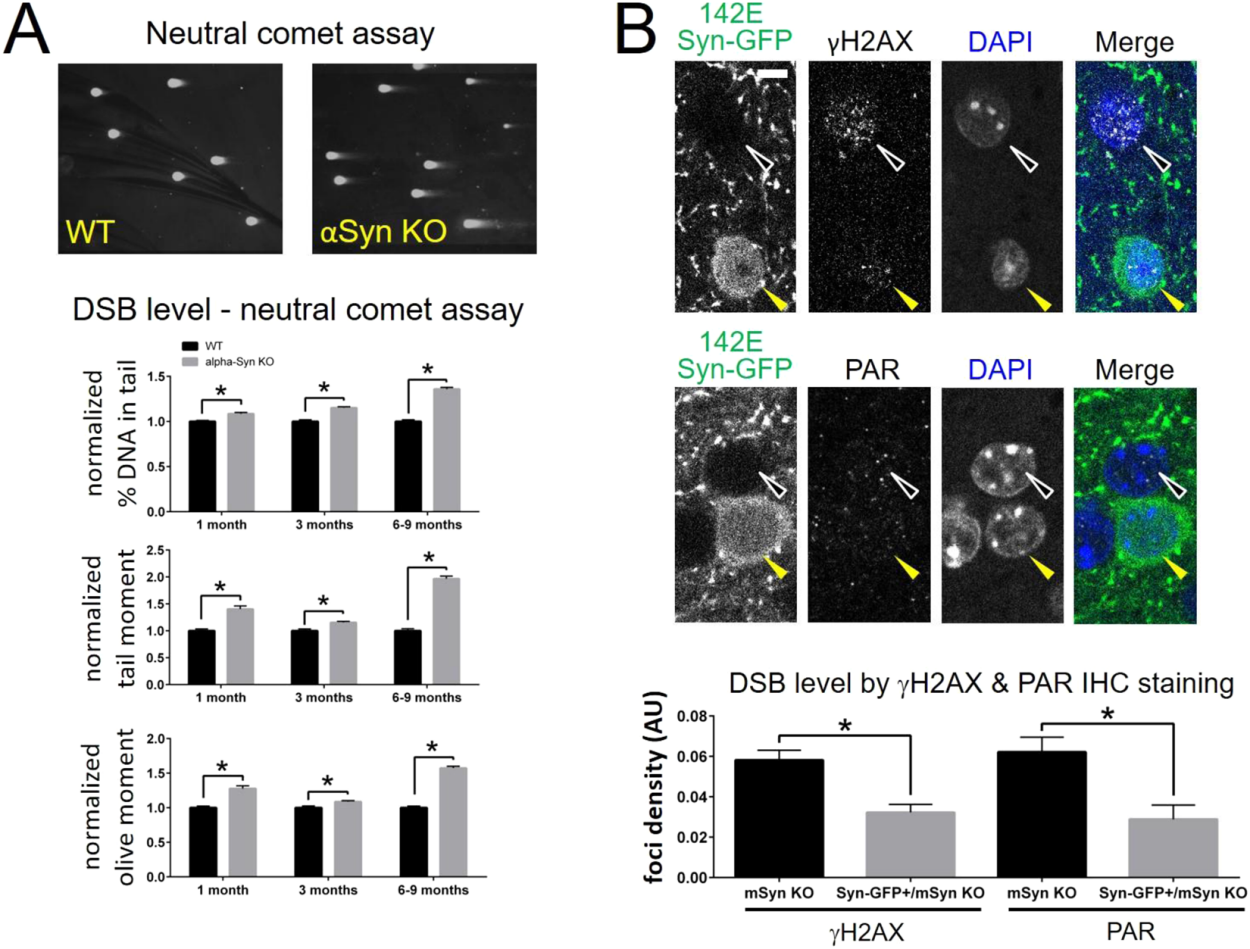
Alpha-synuclein knock-out in mice increases cortical neuron DSBs, which can be rescued by transgenic expression of human alpha-synuclein. (**A**) Neutral comet assay shows increased levels of DSBs in SNCA KO (αSyn KO) mice compared to WT control mice, at 1, 3 and 6–9 months of age (normalized % DNA in tail 1 month-old: αSyn KO = 1.09 ± 0.014, WT = 1.00 ± 0.011, 3 month-old: αSyn KO = 1.15 ± 0.011, WT = 1.00 ± 0.020; 6–9 month-old αSyn KO = 1.36 ± 0.02, WT = 1.00 ± 0.019; tail moment 1 month-old: αSyn KO = 1.40 ± 0.059, WT = 1.00 ± 0.034, 3 month-old: αSyn KO = 1.15 ± 0.022, WT = 1.00 ± 0.035; 6–9 month-old αSyn KO = 1.97 ± 0.048, WT = 1.00 ± 0.038; olive moment 1 month-old: αSyn KO = 1.28 ± 0.041, WT = 1.00 ± 0.024, 3 month-old: αSyn KO = 1.09 ± 0.014, WT = 1.00 ± 0.026; 6–9 month-old αSyn KO = 1.57 ± 0.028, WT = 1.00 ± 0.024; unpaired t-tests for all comparisons p < 0.0001 except for olive moment 3 month p = 0.0012; 1 month-old: WT N = 601 comets/3 animals, αSyn KO = 354 comets/3 animals; 3 month-old: WT N = 183 comets/3 animals, αSyn KO = 291 comets/3 animals; 6–9 month-old: WT N = 683 comets/3 animals, αSyn KO = 822 comets/3 animals). (**B**) IHC for γH2AX (top) and PAR (middle) shows decreased levels of these DDR markers in cells expressing 142E Syn-GFP transgene (yellow arrowhead) on mouse alpha-synuclein (mSyn) KO background compared to cells not expressing 142E Syn-GFP (white arrowhead). Scale bar 2.5 μm. Bottom: Group data (γH2AX foci density mSyn KO = 0.0581 ± 0.0049 AU, N = 16 cells/4 animals; 142E Syn-GFP/mSyn KO = 0.0322 ± 0.0040 AU, N = 77 cells/4 animals, unpaired t-test p = 0.0052; PAR foci density mSyn KO = 0.0622 ± 0.0073 AU, N = 98 cells/4 animals; 142E Syn-GFP/mSyn KO = 0.0288 ± 0.0070 AU, N = 65 cells/4 animals, unpaired t-test p = 0.0021).

In order to further test whether alpha-synuclein modulates DSB repair, we next studied the effect of genetic deletion of alpha-synuclein in SNCA knock-out mice. Using the neutral comet assay to detect and quantify DSB levels in brain tissue, we found a significant increase in DSBs in SNCA knock-out mouse brain compared to WT animals at 1 month-, 3 month- and 6–9 month-old (Fig. 4A). We next tested whether expression of human alpha-synuclein on the mouse SNCA knock-out background could rescue the increased DSB levels in mouse cortical neurons. We measured γH2AX and PAR levels in a transgenic line generated by Masliah and colleagues^32^ in which human alpha-synuclein is tagged on its C-terminus with GFP using a two amino acid linker containing glutamate at the second linker position (142E Syn-GFP). These 142E Syn-GFP animals were then crossed to a mouse SNCA knock-out background. The 142E Syn-GFP transgene is only expressed in a subset of cortical neurons in this line (~2%)^33^, allowing us to compare nuclear γH2AX and PAR levels directly between neurons with mouse alpha-synuclein (mSyn) deleted versus adjacent neurons expressing the 142E Syn-GFP transgene on a mSyn knock-out background. Expression of the 142E Syn-GFP transgene significantly lowered DNA damage levels, as measured by nuclear γH2AX and PAR staining (Fig. 4B). These data suggest that removing alpha-synuclein in mouse brain increases DSB levels, and that this effect can be rescued by addition of the human alpha-synuclein protein, which compensates for the loss of mouse alpha-synuclein in a cell autonomous manner.

### Alpha-synuclein binds double-stranded DNA and facilitates the DNA non-homologous end-joining reaction

**Figure 5:**
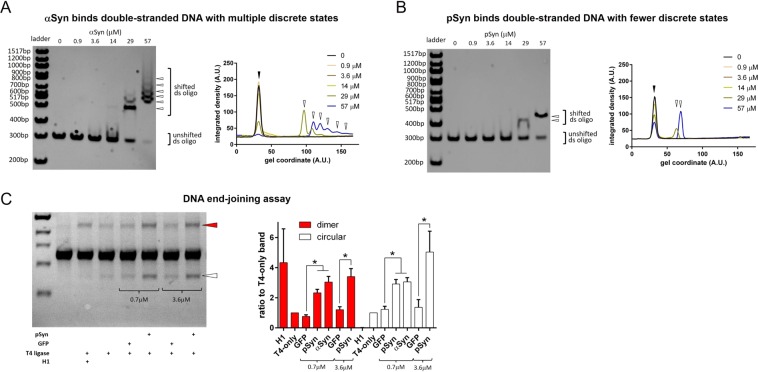
Alpha-synuclein binds double-stranded DNA and facilitates the DNA non-homologous end-joining reaction. (**A**) Left: EMSA of shifted 300 bp dsDNA run on a 6% polyacrylamide gel with increasing alpha-synuclein (αSyn) shows at least 6 different bound states (white arrowheads). Right: Integrated density of each band plotted versus distance on the gel shows progressive reduction of the unshifted peak (black arrowhead) and simultaneous increase in the multiple shifted peaks (white arrowheads) with increasing αSyn concentration. (**B**) Left: Similar EMSA with S129-phospho-synuclein (pSyn) shows only 2 different bound states (white arrowheads). Right: Integrated density of each band plotted versus gel distance shows similar, but fewer, shifted peaks (white arrowheads) with increasing pSyn concentration, also associated with a reduction of the unshifted peak (black arrowhead). (**C**) Left: DNA T4 ligase-mediated non-homologous end-joining assay shows formation of ligation products, including dimer band (red arrowhead) and circularized monomer (white arrowhead), with two concentrations of pSyn compared to GFP (negative control) and histone H1 (positive control). Right: Group data shows a significant difference between control and pSyn and αSyn for both the dimerization and circularized monomer products (dimerization ratio versus T4 ligase-only condition: 0.7 μm protein GFP = 0.76 ± 0.11, N = 6; pSyn = 2.33 ± 0.23, N = 10, αSyn = 3.04 ± 0.37, N = 6, F(2, 19) = 16.77, p < 0.0001, ANOVA, post-hoc Tukey test: GFP vs. pSyn p = 0.0011, GFP vs. αSyn p < 0.0001, pSyn vs. αSyn p = 0.1450; 3.6 μm protein GFP = 1.21 ± 0.19, N = 4; pSyn = 3.40 ± 0.53, N = 7, t-test p = 0.0154; circularization ratio versus T4 ligase-only condition: 0.7 μm protein GFP = 1.23 ± 0.20, N = 6; pSyn = 2.91 ± 0.30, N = 10, αSyn = 3.05 ± 0.28, N = 6, F(2, 19) = 10.77, p = 0.0007, ANOVA, post-hoc Tukey test: GFP vs. pSyn p = 0.0015, GFP vs. αSyn p = 0020, pSyn vs. αSyn p = 0.9349; 3.6 μm protein GFP = 1.21 ± 0.19, N = 4; pSyn = 3.40 ± 0.53, N = 7, t-test p = 0.0007).

Given our findings that alpha-synuclein localizes to sites of DNA damage and that its deletion increases DSB levels, we next set out to test whether alpha-synuclein could directly bind double-stranded DNA and how this might modulate the non-homologous end-joining reaction catalyzed by a ligase enzyme. We used an electrophoretic mobility shift assay (EMSA) assay to test whether alpha-synuclein and the serine-129 phosphorylated (S129-phospho) form of the protein could bind DNA. Increasing amounts of either alpha-synuclein or S129-phospho-synuclein in a 10% polyacrylamide gel system shifted double-stranded DNA to higher apparent molecular weights, which was not seen with a control protein, glutathione-S transferase, at the same concentrations (Fig. 5 Supplemental). We next used a lower concentration, 6% polyacrylamide gel system in similar EMSA experiments. Alpha-synuclein produced a DNA laddering effect, while S129-phospho-synuclein did not (Fig. 5A,B). This suggests that alpha-synuclein interactions with DNA can produce multiple, discrete bound states, while the number of such potential states is reduced by serine-129 phosphorylation. To test the potential functional consequences on DSB repair of direct DNA binding by alpha-synuclein, we used a purified component system where a ~1 kb linear DNA molecule was incubated with T4 ligase at limiting concentrations. A similar assay has been used to show that PC4/SUB1 can directly facilitate DSB repair by promoting the non-homologous end-joining reaction^34^. In this system, both alpha-synuclein and S129-phospho-synuclein were able to enhance the ligation of DNA into either a ~2 kb dimer or the circularized ~1 kb monomer, compared to soluble GFP protein used as a control (Fig. 5C). This mechanism of facilitating DNA end-joining by alpha-synuclein shares some, but not all, aspects with our positive-control protein, the histone H1. H1 is a known enhancer of the dimerization end-joining reaction, but under our experimental conditions it completely suppressed the circularization reaction, while synuclein facilitated both dimerization and circularization (Fig. 5C). This suggests that alpha-synuclein binds DNA, and that this binding may produce a different structural state of DNA than occurs with H1 binding.

### Nuclear alpha-synuclein is rapidly recruited to sites of laser-induced DNA damage *in vivo* and in culture

**Figure 6:**
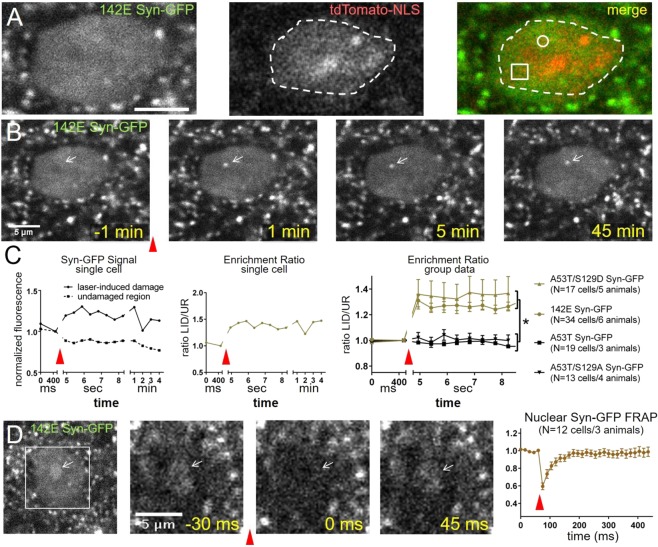
Nuclear alpha-synuclein is rapidly recruited to sites of laser-induced DNA damage *in vivo*. (**A**) Cortical neuron cell body imaged *in vivo* in a mouse expressing 142E Syn-GFP (heterozygous) and nuclear localized TdTomato-NLS (heterozygous). White circle in merge image shows targeting of laser-induced damage (LID) pulse and white square is the control region. Dotted line represents the outline of the nucleus. Scale bar 5 μm. (**B**) Baseline (t = −1min) and after LID (t = 1 min) images show accumulation of Syn-GFP at DNA damage site (white arrow). (**C**) Data from B) shows increased (at LID site) and decreased (at adjacent site, square in A) Syn-GFP level, calculated enrichment ratio at LID site, and group data from different transgenic (142E Syn-GFP & A53T Syn-GFP) and AAV8-mediated expression (A53T/S129D Syn-GFP & A53T/S129A Syn-GFP) animals (142E Syn-GFP Enrichment Ratio = 1.26 ± 0.03, N = 34 cells/6 animals, A53T Syn-GFP Enrichment Ratio = 0.95 ± 0.02, N = 19 cells/3 animals, A53T/S129D Syn-GFP Enrichment Ratio = 1.37 ± 0.13, N = 17 cells/5 animals, A53T/S129A Syn-GFP Enrichment Ratio = 1.00 ± 0.04, N = 13 cells/4 animals; F(3, 78) = 9.086, p = 0.0001, ANOVA, post-hoc Tukey tests: A53T/S129D vs. A53T p = 0.0002, A53T/S129D vs. A53T/S129A p = 0.0051, 142E vs. A53T p = 0.0016, 142E vs. A53T/S129A p = 0.0343, A53T/S129D vs. 142E p = 0.6078, A53T vs. A53T/S129A p = 0.9726). (**D**) White square shows area magnified to the right. FRAP shows rapid mobility of Syn-GFP within LID site (white arrow, τ_recovery_ = 33.1 ms, 95% CI = 23.8–54.4 ms, N = 12 cells/3 animals). Red arrowheads in all sections show time of LID or FRAP laser pulse.

To further test the role of nuclear alpha-synuclein in the DDR pathway, we developed an *in vivo* experimental paradigm that allows us to visualize alpha-synuclein in real time following focal DNA damage. Using multiphoton microscopy through a cranial window, we generated small, subnuclear regions of DNA damage using focused, short laser pulses, and then measured the immediate response of GFP-tagged human alpha-synuclein (Syn-GFP). Laser-induced DNA damage using multiphoton laser illumination has been used extensively to study recruitment of specific proteins involved in DSB repair in cell culture^35–37^, but to our knowledge, has not been translated previously for *in vivo* use in mouse brain. We used multiphoton illumination at ~730 nm wavelength, since previous work has shown that this is able to induce DSBs^38^. We tested the ability of focal DNA damage to recruit Syn-GFP to DSBs in several different point mutation mouse models that we have created, including the human disease-causing mutation (A53T), and substituted residues at amino acid position serine-129 to mimic (S129D) or prevent (S129A) its phosphorylation. We also analyzed the 142E Syn-GFP human synuclein mouse line, where the glutamate (142E) in the two amino acid C-terminal linker potentially mimics the extra C-terminal negative charge of phosphorylation. Focal illumination of small nuclear subregions with a short pulse of high intensity laser light induced the rapid (<4 sec) redistribution of 142E Syn-GFP to the site of damage (Fig. 6A–C). Interestingly, this recruitment appeared to be dependent upon the specific amino acid present at residue 129. The phosphomimetic S129D resulted in rapid recruitment; but alpha-synuclein with alanine at this same position S129A, or wild-type serine did not (all on the A53T background, Fig. 5D). These results suggest that alpha-synuclein is rapidly recruited to sites of focal DNA damage, and that the C-terminal charge state of alpha-synuclein is important for rapid accumulation at damage sites *in vivo*. We next used *in vivo* multiphoton fluorescence recovery after photobleaching (FRAP) approaches^25,33,39^ to measure the mobility of alpha-synuclein recruited to damage sites. In these experiments, 142E Syn-GFP remained rapidly mobile, with a recovery τ = ~30 ms (Fig. 6D) after accumulation at sites of DNA damage, suggesting that it is not irreversibly bound. A similar nuclear localization to laser-induced damage sites was also obtained in a mouse primary cortical neuron culture system with all five different synuclein constructs that we tested (containing S129D or S129A Syn-GFP, with or without the A53T mutation, and 142E Syn-GFP, Fig. 6 Supplemental). These experiments show that when DSBs are induced in mouse cortex *in vivo* or in a primary culture system, fluorescently tagged forms of alpha-synuclein are rapidly recruited to sites of DNA damage.

### Mouse and human Lewy pathology are associated with increased DSBs

**Figure 7:**
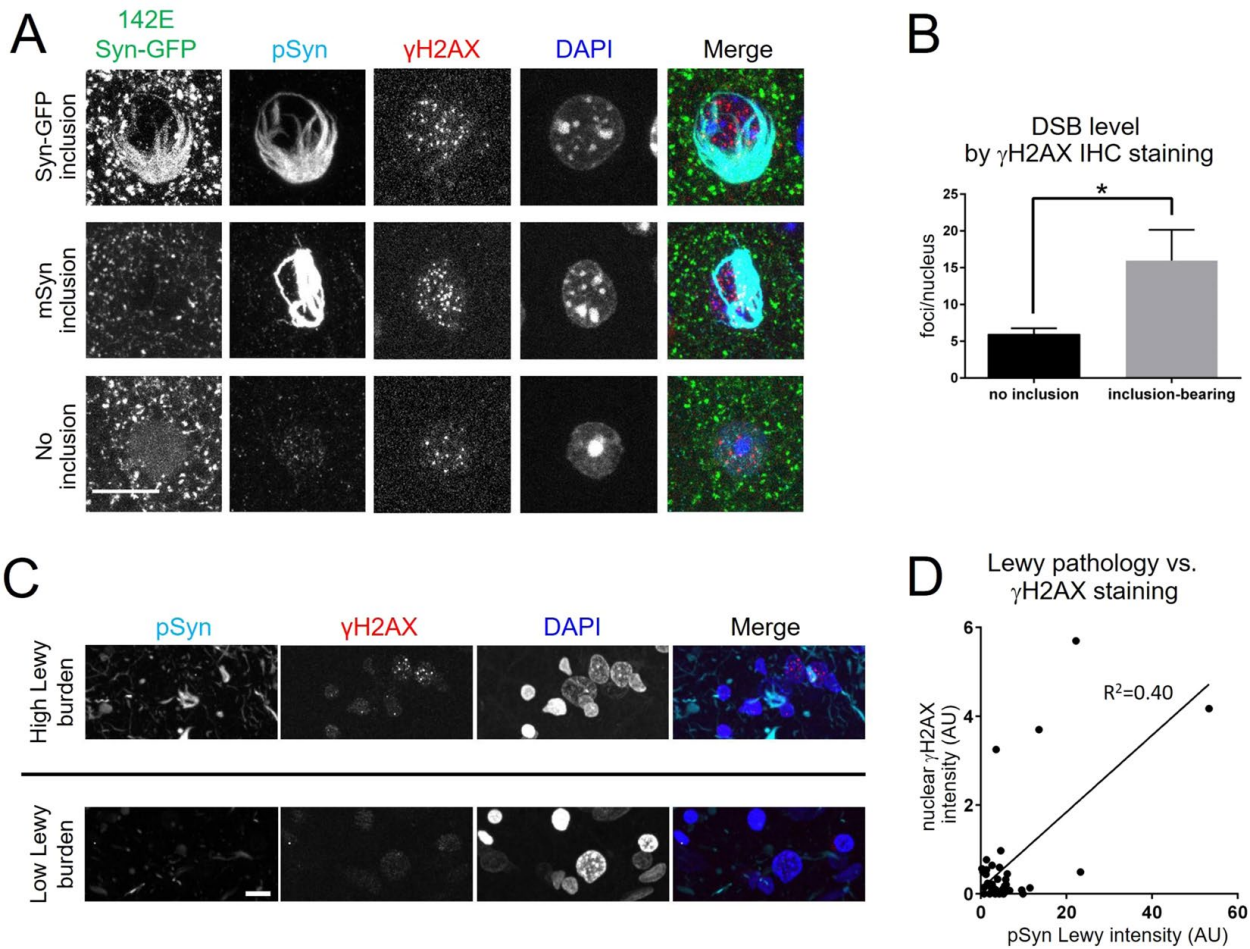
Lewy pathology is associated with increased DSBs in mouse & human cortex. (**A**) Formation of PFF-seeded 142E Syn-GFP-positive or untagged endogenous mouse synuclein (mSyn) Lewy inclusion-bearing neuron in cortical fixed tissue is associated with increased DSB levels compared to adjacent cells with no inclusion. Scale bar 10 µm. (**B**) Group data showing combined (142E Syn-GFP and mouse-only) Lewy pathology-associated DSB levels compared to adjacent cells without Lewy pathology (nuclear γH2AX levels: Lewy inclusion-containing cells = 16.0 ± 4.2 foci/nucleus, N = 23 nuclei/4 animals, cells without pathology = 5.9 ± 0.8 foci/nucleus, N = 23 nuclei/4 animals, unpaired t-test p = 0.0235). (**C**) Human DLB amygdala staining for regions with high (top) and low (bottom) levels of Lewy pathology burden show increased nuclear γH2AX staining in regions with high burden. Scale bar 10 μm. (**D**) Group data showing positive correlation between Lewy pathology burden and DSB levels as measured by nuclear γH2AX staining (normalized to DAPI volume, R^2^ = 0.40, p = 0.0001, N = 40 regions/6 cases).

Given our data suggesting that alpha-synuclein can facilitate DSB repair, we set out to test whether this nuclear function for alpha-synuclein may be relevant to the pathogenesis of synuclein-related disease. Our previous work showed that the induction of Lewy pathology in individual cortical neurons in 142E Syn-GFP mice by intracortical injection of recombinantly produced alpha-synuclein preformed fibrils (PFFs) produces inclusions that are structurally similar to human Lewy pathology. For example, PFF-induced mouse inclusions are composed of fibrillar alpha-synuclein that is ubiquitinated and phosphorylated at S129^25^. Our previous work also detected a dramatic loss (97 ± 6% decrease) of soluble alpha-synuclein from the nucleus of inclusion-bearing neurons, suggesting that soluble synuclein may become trapped in immobile, cytoplasmic Lewy inclusions^25^. To test whether this Lewy inclusion-induced loss of nuclear alpha-synuclein could disrupt DSB repair, here we measured DSB levels using nuclear γH2AX staining in neurons with and without somatic Lewy pathology. Our analysis showed a significant increase in the level of DSBs specifically in inclusion-bearing cells in mouse cortex (Fig. 7A,B), suggesting that nuclear alpha-synuclein loss-of-function, induced by somatic Lewy pathology formation, may lead to increased accumulation of DSBs. This was consistent with our detection of increased DSBs following genetic deletion of alpha-synuclein in mouse brain (Fig. 6A). Induction of Lewy pathology in mouse hippocampal neurons in primary culture by PFF seeding also induced a significant ~6-fold increase in DSBs as compared to control cultures, measured by nuclear γH2AX staining (Fig. 7A Supplemental).

We next asked whether alpha-synuclein pathology correlates with increased DSBs in diseased human brain tissue. We measured levels of Lewy pathology by phosphorylated alpha-synuclein staining, and DSBs by γH2AX staining, in human amygdala tissue from patients with the synucleinopathy Dementia with Lewy Bodies (DLB). Amygdala was chosen because it is a brain region known to have high Lewy pathology burden in DLB. We detected individual neurons containing somatic Lewy bodies that showed increased DSB levels compared to neighboring neurons without Lewy bodies (Fig. 7B Supplemental); however, the frequency of Lewy bodies in regions without simultaneous heavy Lewy neurite pathology (which we cannot assign to individual neurons) was too low to perform a useful analysis. Therefore, we next assessed how regions of amygdala with higher or lower levels of Lewy pathology (both Lewy bodies and neurites) correlated with nuclear γH2AX levels in the same regions. This analysis showed a significant correlation between the overall Lewy pathology burden and DSB level in the amygdala (Fig. 7C–D), suggesting that in human disease, loss of soluble alpha-synuclein through aggregation into cytoplasmic Lewy pathology is associated with increased DSBs. Each of these results suggests that when alpha-synuclein is aggregated into Lewy pathology in the cytoplasm of neurons, which we have previously shown leads to decreases in nuclear levels of the protein^25^, increased levels of DSBs appear, either in culture, mouse cortex or human amygdala.

## Discussion

Although alpha-synuclein’s presence and role in the nucleus has been controversial^4^, we have found clear evidence for its existence in the nucleus in the human HAP1 cell line and in mouse cortical neurons, consistent with previous reports^3,5–7,11–14,40^. Here we demonstrate that endogenous nuclear alpha-synuclein can be found in discrete foci that colocalize with markers of DNA damage in dividing HAP1 cells and post-mitotic mouse cortical neurons (Figs 1 and 1B Supplemental). Using a combination of approaches in HAP1 cells, including ICC, nuclear fractionation, western blotting and the neutral comet assay on WT and SNCA knock-out cells, we found direct evidence that alpha-synuclein plays a role in regulating cellular repair responses to induced DSBs (Fig. 3). Importantly, SNCA knock-out mouse cortical neurons showed increased levels of DSBs and these levels could be reduced by re-expression of human alpha-synuclein-GFP in a cell autonomous manner (Fig. 4). Biochemical studies using EMSA and a DNA non-homologous end-joining assay showed that both alpha-synuclein and S129-phospho-synuclein can bind DNA (Figs. 5 and 5 Supplemental) and facilitate the catalysis of DNA end-joining by T4 ligase (Fig. 5C). We also developed a new, *in vivo* multiphoton assay for inducing DNA damage in individual cortical neurons in living mouse brain. Using this approach *in vivo*, we showed that S129-phospho-mimetic synuclein forms are rapidly and selectively recruited to sites of DNA damage (Fig. 6). Finally, Lewy inclusion-containing neurons in culture, intact mouse cortical tissue, and human patient brain tissue all demonstrated increased levels of the DDR component γH2AX, consistent with increased levels of DSBs in these cells (Figs 7 and 7A,B Supplemental). Together our data suggest a model whereby aggregation into cytoplasmic Lewy bodies may decrease alpha-synuclein’s nuclear function in DNA repair, leading to an accumulation of DSBs that could ultimately trigger cell death in Lewy body-containing neurons in disease.

Our results suggest that nuclear alpha-synuclein plays an important role in regulating cellular responses to DNA damage, including DSBs, a particularly toxic form known to induce programmed cell death^41,42^. Our previous work suggests that soluble alpha-synuclein is greatly reduced in the nucleus and cytoplasm of Lewy inclusion-bearing cells, and that these neurons are much more likely to die than non-inclusion-bearing cells^25^. We now demonstrate that part of the mechanism of cell death in Lewy inclusion-bearing neurons could be due to a loss of nuclear alpha-synuclein’s activity in the DSB repair pathway. This is particularly critical in post-mitotic neurons, where non-homologous end-joining forms of DSB repair are thought to be the only mechanism for repairing DSBs. Our data presented here suggests that alpha-synuclein is an important modulator of non-homologous end-joining forms of DSB repair. Therefore, loss of nuclear alpha-synuclein in Lewy inclusion-containing neurons may compromise DSB repair – causing a loss-of-function of synuclein. This intriguing mechanism must be reconciled with decades of work showing that alpha-synuclein aggregation can lead to toxic gains-of-function for this protein within neurons. For example, alpha-synuclein aggregates can target multiple cellular processes via such gains-of-function within neurons, such as mitochondrial respiration & biogenesis, Golgi-to-ER trafficking, protein homeostasis by autophagy & the ubiquitin-proteasome system, axonal transport, and presynaptic function^43^. In part, the concept that alpha-synuclein loss-of-function could play a role in disease has not garnered as much attention because SNCA knock-out mice do not show a pronounced neurodegenerative phenotype^44^. One possible explanation for the relative lack of neurodegeneration seen after constitutive knockout of alpha-synuclein throughout development is compensation by other genes. The most likely (but by no means the only) candidates for mediating compensatory changes are the other two synuclein family members: beta- and gamma-synuclein. Conflicting data exist about whether SNCA knock-out leads to upregulation of these other family members, with reports showing that targeted SNCA deletion leads to beta-synuclein upregulation^45^; in contrast, in a spontaneously arising alpha-synuclein deletion mutation resulting from a presumably much larger chromosomal deletion (<2 cM), no such upregulation was seen^46^. The potential compensatory interactions between these three genes are likely to be complicated. Combined genetic deletion of SNCA & SNCB leads to gamma-synuclein upregulation^47^, and beta-synuclein transgenic overexpression reduces alpha-synuclein expression^48^. More work needs to be done to test whether depletion of alpha-synuclein from neurons in a regulated fashion in adult animals could reduce compensatory changes and lead to more pronounced neurodegeneration phenotypes that could be explained by alpha-synuclein loss-of-function. Despite this controversy, here we show that even the singular removal of alpha-synuclein leads to increased DSB levels, which may not have been measured in previous studies.

An alternative strategy to knock-out approaches to reduce alpha-synuclein in adult animals is RNAi-mediated knock-down. The data using this approach is also somewhat conflicting, however, with some work reporting that alpha-synuclein knock-down in adult rodents does not induce detectible neuronal toxicity^49,50^. In contrast, others have suggested that knock-down in adult rodents and primates leads to neuronal cell death^51,52^, consistent with a loss-of-function mechanism of toxicity. In addition, it has also been reported that alpha-synuclein knock-down results in mixed positive and negative effects^53,54^. It may be that the magnitude of knock-down is critical to reveal loss-of-function phenotypes. In our work presented here, it is interesting that even in complete SNCA knock-out HAP1 cells at baseline, without addition of another stressor, we did not detect differences in DSB levels compared to WT cells using multiple techniques (Fig. 2). It was only after we induced the formation of DSBs with bleomycin that clear deficiencies in SCNA knock-out cells compared to WT with regards to DSB repair were revealed (Fig. 3). Mouse cortical neurons were different, however, since even at baseline SNCA knock-out cells had higher levels of DSBs (Fig. 4). This suggests that neurons in the brain may be exposed to higher levels of DSB-inducing insults even under baseline conditions. It may be that alpha-synuclein loss-of-function only leads to neurodegeneration when the protein is reduced below specific levels, partly determined by the magnitude of cumulative DNA damaging insults experienced by the cell. Therefore, the critical level of alpha-synuclein below which neurodegeneration is induced may be different depending upon context. Future studies are warranted to try correlating the amount of RNAi-mediated SNCA knock-down with neurodegeneration observed.

The nuclear functions that we have identified for alpha-synuclein in DSB repair place it within the increasing family of neurodegeneration-related proteins that are implicated in DNA and/or RNA binding and DSB repair. For example, the microtubule-associated protein tau is suggested to play a role in regulating neuronal DSB levels^55,56^. Several other neurodegeneration-related proteins also have established DNA binding activity, including TDP-43^57^, TLS/FUS^58^, and TRF2^59^. Although the roles of these proteins in DNA repair require further investigation, the fact that TLS/FUS^60^, TRF2^61^ and now alpha-synuclein (Figs 6, 6 Supplemental) are all recruited within seconds to sites of DSBs using laser-induced DNA damage protocols suggests that this localization may be important in the repair process. In addition, tau, TDP-43, TLS/FUS and TRF2 have all been suggested to cause neuronal toxicity via a nuclear loss-of-function mechanism. We propose that some of the toxicity related to Lewy pathology formation in neurons may also be due to a loss-of-function mechanism related to nuclear DNA repair, making alpha-synuclein’s function more similar to that of tau, TDP-43, TLS/FUS and TRF2 than previously expected.

To our knowledge, a possible link between synucleinopathy and DNA repair has only been suggested in a few situations^22,62^, including within the context of the disease Ataxia-Telangiectasia (AT). AT is due to a loss-of-function of the DSB-repair-related gene Ataxia-Telangiectasia Mutated (ATM). Several human AT cases were reported to have dopamine neuron loss in the substantia nigra and Lewy body pathology^63,64^. The mouse ATM knock-out model also shows a progressive loss of nigral dopamine neurons along with some form of aggregated alpha-synuclein pathology^65^, although it is not clear if these mouse aggregates are true Lewy inclusions. The alpha-synuclein pathology reported in the ATM work is especially interesting in the context of our work, showing that pathological inclusion formation causes alpha-synuclein loss-of-function and increased DSBs, because it suggests a possible bidirectional relationship between DDR dysfunction and alpha-synuclein aggregation – that defects in DSB repair might also cause Lewy pathology formation. Take together these findings suggest that the relationship between alpha-synuclein and DSB repair in neurons may be an intimate one, with many potentially interesting links still to be discovered. Recent work suggests a potential mechanism by which alterations in DNA repair could induce Lewy pathology formation. Kam *et al*. have demonstrated an unexpected link between poly-ADP-ribose (PAR) polymer and the toxicity of alpha-synuclein fibrillar aggregates^66^. In this work, PAR was able to directly bind alpha-synuclein fibrils and encourage further fibril formation in purified systems. Further, PAR greatly enhanced the toxicity of bound alpha-synuclein fibrils, with an increased ability to cause neuronal cell death, both in a culture system and in mouse midbrain dopamine neurons. Although the conditions of our co-immunoprecipitation experiments did not demonstrate direct binding of nuclear alpha-synuclein to γH2AX or PAR within HAP1 cells (data not shown), these two molecules were shown to colocalize in the nucleus (Fig. 1B,D). It is reasonable to suspect that under normal conditions their binding affinity for each other could be relatively weak, making co-immunoprecipitation difficult. We speculate that excessive or abnormal DNA repair might lead to conditions that promote the formation of excessive fibrillar alpha-synuclein aggregates bound to PAR within nuclear DNA repair foci. This could be the original source for alpha-synuclein fibril-bound PAR aggregates that then enter the cytoplasm, where they could seed cytoplasmic Lewy inclusion formation in this cell, or which could be transported to other cells, furthering Lewy pathology propagation from cell-to-cell.

All members of the synuclein gene family, including beta- and gamma-synuclein, share similar domain structures with an N-terminal positively charged membrane-binding domain and C-terminal negatively charged acidic amino acid-rich region. The specific nuclear role that we have discovered for alpha-synuclein in the DDR could extend to beta- and gamma-synuclein as well. This could explain why members of the synuclein family are found in non-neural cells, where they are not involved in synaptic transmission. For instance, synuclein family members are upregulated in certain cancers: alpha-synuclein in melanoma^67^; and gamma-synuclein in breast^68,69^, prostate^70^, bladder^71^, uterine^72^ and ovarian^73^ cancer. Since we postulate that alpha-synuclein’s normal nuclear DNA binding and DDR functions may be conserved amongst the other family members, it will be important to test whether the synucleins could also be participating in the DDR in these forms of cancer.

Our data suggest a new framework for understanding the importance of alpha-synuclein in certain forms of neurodegeneration. We have shown that alpha-synuclein is rapidly recruited to sites of DSBs, where it may regulate the DDR and promote DNA repair. We suggest that in Lewy body forms of neurodegeneration, cytoplasmic aggregation of alpha-synuclein reduces soluble nuclear alpha-synuclein levels, potentially inducing a loss-of-function that causes increased DSBs and leading to neuronal programmed cell death. We also postulate that this previously unknown set of functions for alpha-synuclein in DSB repair may extend across synuclein family members. Further understanding of the synuclein protein family’s DNA repair functions could help facilitate the development of new therapeutic targets for several important forms of neurodegeneration and cancer.

## Materials and Methods

### Humans

Human subject tissue was procured through the Oregon Alzheimer’s Disease Center (ADC) and OHSU Department of Pathology and affected subjects had established clinical and pathological diagnoses (Dementia with Lewy Bodies). Tissue use was approved by the IRB at OHSU and performed in accordance with relevant guidelines. Informed consent was obtained from all participants and/or their legal representatives.

### Animals

Animals were housed by OHSU’s Department of Comparative Medicine in a light-dark cycle and temperature- and humidity-controlled vivarium and maintained under ad libitum food and water diet. All experiments were approved by the OHSU IACUC, all experiments were performed in accordance with the relevant guidelines and regulations, and every effort was made to minimize the number of animals used and their suffering.

### Mouse model generation

#### Transgenic mouse lines

Utilizing the OHSU Transgenic Mouse Model Core, we created a mouse expressing human alpha-synuclein fused to enhanced GFP (C-terminal tag) containing a point mutation at Alanine 53 (GCA > ACA) causing a threonine amino acid change (A53T Syn-GFP). The A53T-Syn-GFP sequence was cloned into the MoPrp.Xho vector (gift of David Borchelt)^74^ at the XhoI site. Expression is under transcriptional control of the mouse prion protein promoter. The 30 bp linker sequence between A53T Syn and GFP is GGTACCGCGGGCCCGGGATCCATCGCCACC, which translates to GlyThrAlaGlyProGlySerIleAlaThr. This mouse line shows A53T Syn-GFP expression in >90% of cortical neurons and <10% of astrocytes (A.J.S. and V.K.U., unpublished data). 142E Syn-GFP transgenic mice^32,33,39,75^ and 142E Syn-GFP/mouse mSynKO mouse lines were obtained from the line’s creators, Edward Rockenstein and Eliezer Masliah. Nuclear localized TdTomato-NLS transgenic mice were obtained from the Jackson Laboratories (stock# 023035). Alpha-synuclein knock-out animals and appropriate control mice were obtained from Jackson Laboratories (stock# 016123, 005304).

#### AAV8 virus generation

We created viral constructs of A53T Syn-GFP containing point mutations in alpha-synuclein at serine-129 causing either an alanine (TCT > GCT) or aspartic acid (TCT > GAT) amino acid change (constructs gift of Pamela McLean). These constructs were cloned into the self-complementary AAV8 viral vector pTRS-KS/CBh-GFP (from the National Gene Vector Biorepository, Indianapolis, IN), under transcriptional control of the CBh (Chicken Beta Actin Short) promoter, using the restriction enzyme sites AgeI and ApaI. The 30 bp linker sequence between alpha-Syn & enhanced GFP is GGTACCGCGGGCCCGGGATCCATCGCCACC. The final scAAV8-CBh-A53T-S129A-Syn-GFP and scAAV8-CBh-A53T-S129D-Syn-GFP viruses were made by the Gene Transfer Vector Core at Massachusetts Eye and Ear Infirmary, Boston, MA.

#### Intraventricular AAV8 viral injections

ICV into P0 neonates were done following the free hand protocol^76^. We injected 2 µL of undiluted virus into each lateral ventricle at titers ~1E12 genomic copies per milliliter. The only deviation from this published protocol is that we did not dilute virus in trypan blue.

### Mouse Lewy pathology induction

2 to 3 month-old male mice were injected with mouse WT sequence PFFs according to our previously published protocols^25^. 2.5 μl (2 mg/ml) freshly sonicated PFFs or 2.5 μl PBS was injected into right hemisphere primary sensory-motor cortex in isoflurane (1% to 2%)-anesthetized animals and returned to their home cage. They were sacrificed and prepared for IHC as described below after post-injection interval of 3–6 months (6–9 month-old).

### Mouse brain *in vivo* imaging & analysis

Cranial window surgery and imaging was done using the same protocol as we have previously published^25,39^ in isoflurane anesthetized animals, using a Zeiss LSM 7MP multiphoton microscope outfitted with dual channel BiG (binary GaAsP) detectors and a Coherent Technologies Chameleon titanium-sapphire femtosecond pulsed laser source (tuned to 860 nm for imaging Syn-GFP). Zeiss Zen 2011 image acquisition software was used. For nuclear laser-induced damage (LID) experiments, the Bleaching function in Zen was used to illuminate small, submicron-sized regions within the nucleus with Chameleon laser tuned to ~730 nm for <1 ms). There is a ~4 sec time delay required to switch the laser to and from the LID (~730 nm) wavelength. In a subset of experiments, animals were generated that expressed Syn-GFP simultaneously with nuclear localized TdTomato-NLS. These demonstrated that localizing the LID pulse to regions >2 μm from the periphery of the cell were always positioned within the nucleus. This criterion was used to localize the LID pulse within nuclei of Syn-GFP expressing cortical neurons *in vivo*. LID images were analyzed with ImageJ similarly as to our previously described FRAP experiments^25,39^. Regions of interest (ROIs) were selected to obtain mean fluorescence values in LID and control ROIs within the nucleus. The ratio of the signal at each time point from the LID versus the control ROIs was used to calculate the Enrichment Ratio. For FRAP experiments after LID, a similar photobleaching and analysis protocol was used as our previously published work^25,39^. Data were analyzed in Prism 6 (GraphPad) to obtain single exponential fits to the recovery time course and the immobile and mobile fractions. All animals used were 5 to 9 month-old.

### Mouse cortical neuron cultures and imaging

C57/BL6 mice were used to generate primary neuronal cultures isolated from embryonic mice, based on the methods of Kaech and Banker^77^, and adapted from Gray and colleagues^78^. Briefly, embryos were harvested at 18 days of gestation from anesthetized females. Cortex was dissected, gently minced, and trypsinized to generate suspensions of dispersed neurons. Freshly isolated cortical neurons were electroporated with plasmids encoding two human alpha-synuclein constructs tagged with an enhanced GFP (scAAV-huSyn_s129A:EGFP, scAAV-hySyn_s129D:EGFP). 330,000 electroporated cortical neurons from each construct were plated onto dishes containing poly-L-lysine-coated coverslips in MEM medium (GIBCO/Life Technologies), 5% FBS (Atlanta Biologicals), and 0.6% glucose (Sigma-Aldrich). After 4 hrs, the medium was removed and replaced with Neurobasal Medium supplemented with 1 × GlutaMAX (GIBCO/Life Technologies) and 1× GS21 (MTI-GlobalStem). Each dish was fed every week with 0.5 ml Neurobasal media plus GlutaMAX and GS21, with the first feed (at 5 days *in vitro* (DIV)) containing AraC. After 7 DIV, coverslips from cultured neurons from each construct were imaged using a live cell system with a closed imaging chamber. Cell culture nuclear LID experiments were carried out on the same imaging platform using the same protocols for imaging and analysis as our *in vivo* nuclear LID experiments described above.

### Mouse hippocampal cultures & PFF seeding

Mouse hippocampal cultures and PFF seeding induced Lewy pathology formation was carried out using our previously described techniques^79,80^. Briefly, primary neuronal cultures were prepared from E16-E18 CD1 mouse brains (Charles River). All procedures were performed according to the NIH Guide for the Care and Use of Experimental Animals and were approved by the University of Pennsylvania Institutional Animal Care and Use Committee. Dissociated hippocampal neurons were plated onto poly-D-lysine coated coverslips (Carolina Biological Supply) at a density of ~50,000 cells/cm^2^. Recombinant human alpha-synuclein PFFs bearing the pathogenic S87N mutation^81^ were diluted in PBS at 0.1 mg/mL, sonicated, and diluted in neuronal media. Neurons were treated at 5 ug/ml at DIV 7 and incubated for 12 days. Cells were fixed in 4% PFA/4% sucrose for 15 min at room temperature and then permeabilized in 0.3% Tx-100 and blocked for 1 hour at room temperature in 3% FBS/3% BSA. Primary antibodies (anti-α-Synuclein PhosphoSer129 clone 81 A, 1:2000 dilution, Biolegend cat#825701; Anti-phospho-Histone H2A.X (Ser139) clone JBW301, 1:500 dilution, Millipore cat#05–636) were diluted in the blocking buffer and incubated at room temperature for 3 hours. Coverslips were then washed with PBS 3 times and incubated with the appropriate Alexa-fluor labeled secondary antibodies. Washed coverslips were mounted on slides using Fluoromount-G with DAPI (Fisher Scientific).

Secondary antibodies: Alexa 488, goat anti mouse IgG2a, 1:1000 dilution, Fisher Scientific; Alexa 594, Goat anti mouse IgG1, 1:1000 dilution, Fisher Scientific.

A PE lamina Scanner was used to image coverslips. HALO software (Indica Labs) was used to count γH2AX foci and DAPI stained nuclei located 1.5 mm from the edge of each coverslip. Statistical Analysis of the data was done using Graphpad Prism Version 4.

### Immunofluorescence & analysis

#### HAP1 cells

Hap1 parental WT control (item #C631 batch 29663) and Hap1 Human SNCA 103 bp deletion knockout (item #HZGHC003210c003 batch 2) cell lines were obtained from Horizon Discovery and grown in IMDM media (Gibco# 11995-065) + 10% Fetal Bovine Serum + Pen-Strep and grown in a humidified incubator at 37 C with 5% CO_2_. Cells were seeded on PLL coated #1.5 coverslips in 35 mm dishes one day prior to fixation and grown to ~80% confluency. Cells were fixed in 4% PFA in PBS for 10 minutes at room temperature (RT), washed 1x in PBS and stored at 4 C in PBS until staining procedure. Fixed cells were permeabilized with 1 mL PBS + 0.25% Triton-X 100 shaking gently at RT for 20 minutes. Solution was removed and cells were blocked for 20 minutes in 1 mL Blocking Buffer (0.1% Triton-X 100 10% normal goat serum in PBS). Primary antibodies were diluted in Incubation Buffer (1:5 dilution of Blocking Buffer in PBS) and incubated overnight at RT with gentle shaking. The next day, cells were washed 3x in PBS for 15 minutes. Complementary fluorescently tagged secondary antibodies were diluted 1:1000 in Incubation Buffer, added to cells, and incubated at RT, in the dark overnight, with gentle shaking. The next day, cells were washed 3x in PBS for 20 minutes. Nuclear staining was done just prior to the final PBS wash with 2.5ug/mL DAPI (Sigma D9542) in PBS for 20 minutes. Before mounting, cells were washed briefly in deionized H_2_O. Coverslips were mounted with 13 µL CitiFluor CFMR2 Antifadent Solution and sealed with Biotium CoverGrip Coverslip Sealant. Slides were imaged on a Zeiss Elyra PS.1 710 laser-scanning confocal microscope with Zen software. Z-stacks of 0.5 µm steps were acquired at 63x zoom1, with care taken to never saturate antibody signal within the nucleus.

Primary antibodies used were: anti-Syn1, 1:500 dilution, mouse monoclonal, BD Biosciences, cat. 610786; anti-Poly(ADP-Ribose) Polymer clone 10 H, 1:500 dilution, chicken polyclonal, Tulip BioLabs, cat. 1023; anti-Phospho-Histone H2A.X, 1:500 dilution, rabbit monoclonal, Cell Signaling, cat. 9718; anti-phosphoS129-Syn EP1536Y, 1:500 dilution, rabbit monoclonal, Abcam, ab51253; anti-phosphoS129-Syn 81 A, 1:667dilution mouse monoclonal, Covance, cat. 825701; anti-Syn 4B12, 1:500 dilution, mouse monoclonal, Biolegend, cat. 807804; and anti-Syn EPR20535,1:100 dilution, rabbit monoclonal, Abcam, ab212184. Secondary antibodies used were: Alexa Fluor 647 goat anti-rabbit, ThermoFisher cat.A-21245; Alexa Fluor 555 goat anti-mouse, Abcam ab150114; Alexa Fluor 488 donkey anti-chicken, Jackson ImmunoResearch cat.703-545-155.

Bleomycin treatment ICC: Bleomycin Sulfate (Selleckchem, cat. S1214) powder was diluted in H_2_O to make a 10 mg/mL stock solution, aliquoted and stored at −80C. One day before treatment Hap1 WT and Hap1 SNCA KO cells were seeded on PLL coated #1.5 coverslips in 35 mm dishes to be ~80% confluent the following day. The day of treatment, normal media was removed and 2 mL fresh warm media containing 10ug/mL Bleomycin was added to each dish. Sham cells had fresh warm media, without Bleomycin, added to each dish. Cells were placed in a humidified incubator for 1 hr at 37 C with 5% CO_2_. After treatment, the media was removed and the cells were fixed in 4% PFA in PBS for 10 minutes at RT, washed 1x in PBS and stored at 4 C in PBS until staining procedure.

Bleomycin treatment Western Blot: One day before treatment Hap1 WT and Hap1 SNCA KO cells were seeded on 2 × 10 cm plates per condition to be ~80% confluent the following day. Bleomycin treatment was done the same as for ICC. After treatment, the media was removed and cells were washed 1x with warm PBS. Cell were harvested by trypsinization and collected into 15 mL conical tubes pelleted for 5 min 200rcf. Liquid was aspirated, pellets were resuspended in 2 mL PBS and transferred to 2 × 2 mL microcentrifuge tubes. Proteins were extracted into cytosolic and nuclear fractions using the NE-PER extraction kit (Thermo-Fisher, cat. 78833) according to the manufacturer’s recommendations with the addition of a brief sonification (10 seconds, 10 kHz) after the first nuclear resuspension step. Protein preps were stored at −80C until Western blot analysis.

For confocal imaging, images were acquired on a Zeiss Elyra PS.1 confocal microscope with a Plan-Apochromat 63x/1.40 oil objective. All colocalization analysis was done using ImageJ (NIH) software by create ROIs for each nucleus from a single image located in the middle of the nucleus in the z direction. The CoLoc 2 plugin was used to measure the Pearson’s coefficient between signals and the expected background value after random translation of one channel versus the other.

#### Mouse brain

Male mouse brains (age 3–6 month-old) were dissected immediately post mortem, placed into 6 mL of fresh 4% PFA in PBS, and fixed with a Pelco Biowave Pro for 90 minutes at 150 W in a circulating water bath. The brains were moved to 4 °C to continue to fix overnight. The next day the PFA was replaced with 0.05% sodium azide and stored at 4 °C until further processing.

After fixation, the brains were sliced into 50 µm coronal or sagittal floating sections using a Vibratome Leica VT1000S. For certain antibodies, heat induced epitope retrieval (HIER) was required prior to blocking. The brain slice was added to a tube containing HIER buffer (1 mM EDTA, 10 mM Tris Base, 0.05% Tween, pH = 8.5), steamed for 20 min, and cooled at room temperature for 20 min. Tissue was blocked for 1 hour in Blocking buffer (0.1% Triton-X, 10% goat serum, in PBS). Primary antibody was diluted in incubation buffer (1:5 dilution of blocking buffer) at a concentration optimized for each antibody and incubated overnight, in the dark, while shaking at room temperature. The tissue was washed for 30 min with PBS 5 times. The complementary secondary antibody was diluted in incubation buffer and incubated similarly overnight at room temperature. The next day, the tissue was washed with 5 exchanges of PBS. DAPI staining was done just prior to the final wash. The tissue was mounted onto a slide in CitiFluor CFMR2 Antifadent Solution. A #1.5 coverslip was sealed over the tissue with Biotium CoverGrip Coverslip Sealant.

Primary antibodies used were: anti-α-Synuclein Phospho (Ser129) Antibody clone 81 A, mouse monoclonal, 1:667 dilution, Biolegend cat#825701; Anti-alpha Synuclein (phospho S129) antibody [EP1536Y], rabbit monoclonal, 1:500 dilution, Abcam cat#ab51253; Anti-asyn (Syn1/mSyn), mouse monoclonal, 1:500 dilution, BD Biosciences cat#610786; Anti-pan-ADP-ribose binding reagent, rabbit, 1:1000 dilution, Millipore cat#MABE1016; anti-Poly(ADP-Ribose) Polymer, clone 10 H (PADPR), chicken, 1:100 dilution, Tulip BioLabs cat#1023; Anti-phospho-Histone H2A.X (Ser139) Antibody clone JBW301, mouse, 1:500 dilution, Millipore cat#05-636; Anti-phospho-Histone H2A.X (Ser139) Antibody clone 20E3, rabbit monoclonal, 1:500 dilution, Cell Signaling cat#9718 Secondary antibodies used were: Alexa 647, goat anti-rabbit, 1:1000 dilution, Invitrogen cat#MPA21245; Alexa 647, goat anti-mouse, 1:1000 dilution, Invitrogen cat#MPA21236; Alexa 555, goat anti-rabbit, 1:1000 dilution, Abcam cat#ab150078; Alexa 555, goat anti-mouse, 1:1000 dilution, Abcam cat#ab150114; Alexa 488, goat anti-chicken 1:1000 dilution, Abcam cat# ab150169.

For confocal imaging, images were acquired on a Zeiss Elyra PS.1 confocal microscope with a Plan-Apochromat 63x/1.40 oil objective. Laser powers of 1–5% were used to acquire *z*-stacks through 2–4 regions of cortex per tissue section. Imaris (Bitplane, Oxford Instruments) imaging software was used to analyze the IHC data. In order to segment out the nucleus from the cytoplasm, a surface was created using the DAPI channel. Only whole neuronal nuclei were analyzed. Statistical information (Mean intensity, Sum intensity, Total number of surfaces) for each channel was exported and further analyzed in Prism 6 (GraphPad). For analysis of foci within the nuclei, the nuclear signal of the channel of choice was masked using a nuclear surface created from the DAPI channel. From the nuclear signal of the desired masked channel, surfaces were made to quantify nuclear foci.

#### Human brain

Sections from the amygdala of human Dementia with Lewy Body autopsy cases were retrieved immediately at autopsy (post-mortem interval 12–48 hours), placed into 6 mL of fresh 4% PFA in PBS, and fixed with a Pelco Biowave Pro for 90 minutes at 150 W in a circulating water bath. The sections were moved to 4 °C to continue to fix overnight. The next day the PFA was replaced with 0.05% sodium azide and stored at 4 °C until further processing.

After fixation, amygdala sections were sliced into 50 µm floating sections using a Vibratome Leica VT1000S. Tissue was blocked for 1 hour in Blocking buffer (0.1% Triton-X, 10% goat serum, in PBS). Primary antibody was diluted in incubation buffer (1:5 dilution of blocking buffer) at a concentration optimized for each antibody (see below) and incubated overnight, in the dark, while shaking at room temperature. The tissue was washed for 60 min with PBS 5 times. The complementary secondary antibody was diluted in incubation buffer and incubated similarly overnight at room temperature. The next day, the tissue was washed with 5 exchanges of PBS. DAPI staining was done just prior to the final wash. The tissue was mounted onto a slide in CitiFluor CFMR2 Antifadent Solution. A #1.5 coverslip was sealed over the tissue with Biotium CoverGrip Coverslip Sealant.

Primary antibodies used were: anti-α-Synuclein Phospho (Ser129) Antibody clone 81 A, mouse monoclonal, 1:667 dilution, Biolegend cat#825701; Anti-phospho-Histone H2A.X (Ser139) Antibody clone 20E3, rabbit monoclonal, 1:500 dilution, Cell Signaling cat#9718. Secondary antibodies used were: Alexa 647, goat anti-mouse, 1:1000 dilution, Invitrogen cat#MPA21236; Alexa 488, goat anti-rabbit, 1:1000 dilution, Abcam cat#ab150077.

Images were captured on a Zeiss LSM 880 with Airyscan microscope. To determine the specific antibody signals from the increased autofluorescent background staining within the human tissue samples, a linear unmixing protocol was used. Lambda mode was used to set up parameters for the objective, lasers and dichroics as required to image the multicolor sample. Using the same parameters, individual reference spectra were created from single color stained samples. In addition, an unstained sample was used to determine the background reference spectra. All residuals were minimized when creating reference spectra. The multicolor sample was then imaged, specifying the relevant background and single-color spectra, and unmixed using Zeiss Zen software.

Imaris (Bitplane, Oxford Instruments) imaging software was used to analyze IHC data. In order to segment out the nucleus from the cytoplasm, a surface was created using the DAPI channel. Only whole neuronal nuclei were analyzed. Statistical information (Mean intensity, Sum intensity, Total number of surfaces) for each channel was exported and further analyzed in Prism 6 (GraphPad). For analysis of foci within the nuclei, the nuclear signal of the channel of choice was masked using a nuclear surface created from the DAPI channel. From the nuclear signal of the desired masked channel, surfaces were made to quantify nuclear foci.

### HAP1 cell comet assay

HAP1 cell neutral comet assays were performed according to Trevigen’s recommendations (Trevigen comet assay kit, cat#4250-050-K). Cell suspensions (2 × 10^4^ cells/ml) in serum-free IMDM (Sigma) mixed at a 1:2 ratio with Comet LMAgarose (Trevigen) were pipetted onto CometSlides^TM^ (Trevigen) and placed at 4 °C for 10–15 min to cast. Slides were incubated overnight in Lysis Solution (Trevigen) at 4 °C in the dark. and then in neutral electrophoresis buffer (300 mM sodium acetate, 100 mM Tris–HCl, pH 9) for 1 hr at 4 °C in the dark. Slides were electrophoresed in fresh buffer for 40 min at 20 V (1 V/cm) at 4 °C. Following electrophoresis, slides were incubated first in DNA precipitation buffer (1 M ammonium acetate, 86.6% EtOH), and then 70% EtOH at room temperature in the dark, for 30 min each and dried at 37 C. Slides were stained with 1X SYBR^TM^green (Invirogen) in 1X PBS (Gibco) at room temperature for 30 min in the dark, dipped in dH_2_O 10 times to wash excess stain, and dried at 37 °C. Comets were visualized on a Zeiss Apotome using a 10X lens and scored using CometScore^TM^.

Bleomycin treatment Comet Assay: One day before treatment Hap1 WT and Hap1 SNCA KO cells were seeded on 2 × 35 mm plates per condition to be ~80% confluent the following day. Bleomycin and Sham treatment protocols were done the same as for ICC. After treatment, the media was removed and cells were washed 1x with warm PBS. If a recovery period followed, then fresh warm media was added and cells were placed back in the incubator. Cells were harvested by trypsinization with 0.05% Tripsin-EDTA (Gibco cat. 15400-54). Trypsinized cells from 2 × 35 mm plates were collected into 15 mL conical tubes and pelleted for 5 min 200rcf. Liquid was aspirated, pellets were resuspended in 250uL fresh media without FBS or Penn-Strep, transferred to a 2 mL microcentrifuge tube and stored on ice until use in the Comet Assay as described above.

### Mouse brain tissue comet assay

Freshly extracted brain tissue from male mice (ages 1, 3, 6–9 months-old) was finely chopped with a razor blade and suspended in ice cold PBS (without Ca^2+^ and Mg^2+^) and homogenized for 30 seconds using a hand-held homogenizer. Tissue was micro-centrifuged for one minute and supernatant diluted 1:10 in PBS. Cells were counted using a hemocytometer and diluted to 1 × 10^5^/ml. Diluted cells were combined with low melting (LM) Agarose at a 1:2 (v/v) ratio and spread over the well of the glass comet slide (Trevigen comet assay kit, cat#4250-050-K). Slides were dried in the dark at 4 °C for 15 minutes and were placed in kit lysis solution at 4 °C overnight. Excess buffer was drained from the slides before immersion in 50 mL TBE, pH 7.4, for 30 min at 4 °C. Slides underwent electrophoresis in TBE, pH 7.4, at 20 V for 40 minutes at 4 °C. Excess TBE was drained and slides were placed in dH_2_O twice for 5 min. Slides were then placed in 70% EtOH for 5 min and dried at 37 °C for 15 min. 100 µl of 1X SYBR-Green were pipetted onto each circle of dried agarose and samples were stained for 30 min at room temperature in the dark. Excess SYBR-Green solution was removed and slides were rinsed in dH_2_O several times. Slides were allowed to dry completely at 37 °C and stored at 4 °C. Comets were visualized on a Zeiss ApoTome microscope under a 10X objective and scored using CometScore™ software (TriTek).

### Electrophoretic mobility shift assay

#### DNA preparation

300 bp fragments were extracted from a 100 bp ladder (NEB) separated on a 1% agarose gel at 175 V for 2 hours at RT using the QIAquick Gel Extraction Kit (Qiagen). DNA was cleaned using the DNA Clean and Concentrator Kit (Zymo Research), and eluted in 1X TE. Final concentration and DNA purity were determined by Nanodrop.

#### Electrophoretic mobility shift assay

DNA fragments (20 ng) were mixed with recombinant hu_ser129_phospho-syn (250 ng, 1 µg, 4 µg, 8 µg, and 16 µg; Proteos, Inc.), recombinant hu_α-synuclein (250 ng, 1 µg, 4 µg, 8 µg and 16 µg; Proteos, Inc.) or recombinant glutathione S-transferase (250 ng, 1 µg, 4 µg, 8 µg and 16 µg; GenScript) in a 20 μL reaction containing 94 mM Tris-Cl, pH 8.0, 25 mM NaCl, 1 mM EDTA, and 5% ficoll on ice for 20 min, then room temperature for 30 min. After addition of 2 µL 10X Orange Loading Buffer (Licor), 8 μL of total reaction were loaded into a 10% or 6% polyacrylamide TBE gel (Novex) and run at 100 V for 2 hours at RT. Gels were stained for 30 minutes with 10X SYBR^TM^ Safe DNA Stain (Invitrogen) in 1X TBE. Images were acquired using the Fluorchem M imaging system and quantified using ImageJ Gel Analyzing tool.

#### Western blot

10% polyacrylamide TBE gels were transferred onto a Biodyne™ B Nylon Membrane (Thermofisher Scientific) at 30 V for 1 hour and 16 min on ice in 0.5X TBE using the Novex XCell II Blotting System (Invitrogen). Membranes were blocked overnight in Odyssey PBS Blocking Buffer (Li-Cor) and stained for 1 hour at RT with Syn1 (1:1,000; Biolegend) and overnight at 4 °C with IRDye^®^ 800CW Goat anti-Mouse IgG (1:10,000; Li-Cor). Images were acquired using Li-Cor Odyssey CLx Imaging System.

Western Blot for Hap1 Nuclear protein preps: Concentrations of the Hap1 NE-PER cytoplasmic and nuclear protein preps were quantified utilizing the Pierce BCA Protein Assay kit (Pierce, cat. 23225) and read on a Multiskan FC Microplate Photometer (Fisher, cat. 51119000) with a 550 nm readout. 5 µg & 10 µg of nuclear protein was diluted in SDS sample buffer (Novex, cat. LC2676) + BME (Gibco, cat. 21985-023) and loaded onto a 10–20% Tris-Glycine gel (Novex, cat. XP10202BOX). 3 µL of color prestained protein ladder (NEB, cat. P7719) was loaded for reference. The gel was ran using the Novex Xcell Blotting System in SDS running buffer (Novex, cat. LC2675) for 70 min at 120 V. The gel was dissembled and set up for transfer onto an Immobilon-FL PVDF membrane (Millipore, cat. IPF00010). The gel was transferred in Tris-Glycine transfer buffer (Novex, cat. LC3675) on ice for 2hrs at 25 V. After transfer, the membrane was removed and immediately fixed in 4% PFA + 0.01% Glutaraldehyde for 10 minutes with gentle shaking at RT. The membrane was washed 1x in milliQ H_2_O and stained with the REVERT total protein stain kit (LI-COR, cat. 926–11010) according to the manufacturer’s protocol. Total stained protein was acquired using the LI-COR Odyssey CLx Imager. After reversal of REVERT stain, the membrane was washed 1x in milliQ H_2_O and blocked in Odyssey Blocking Buffer (LI-COR, cat. 927–40000) overnight at 4 C with gentle shaking. Primary antibodies were diluted 1:1000 in Blocking Buffer and incubated for 2 hr at RT with gentle shaking. The membrane was washed 3x in 0.1% Tween20 in PBS for 10 min. Secondary antibodies (LI-COR IR680LT donkey anti-rabbit cat. 926–68023; IR800CW donkey anti-mouse cat. 926–32212) were diluted 1:10000 in blocking buffer and incubated for 1 hr at RT with gentle shaking. The membrane was washed 3x in 0.1% Tween20 in PBS for 10 min and 1x in PBS for 10 min, at RT. Images were acquired using the LI-COR Odyssey CLx Imager. Analysis was done on FIJI using the gel analyzer. Antibody signal was normalized to REVERT total protein.

### T4 ligase-mediated DNA end-joining assay

Each end-joining reaction contains 150 ng of a 1.2 kb DNA PCR product digested on the 5’ and 3’ ends with cohesive end restriction enzyme XhoI (NEB RO146S). Reactions are carried out in 20 µL reaction volume containing 150 ng digested DNA, 2.8 µL protein buffer (10 mM Tris, 50 mM NaCl pH = 7.6), 5.3 µL 20% glycerol in PBS + Mg + Ca, 2 µL T4 ligase buffer, 200 ng or 40.5 ng protein, 25U of T4 ligase (NEB M0202), and sterile H_2_O to volume. All components except the T4 ligase were combined and incubated on ice for 15 min. 25U T4 ligase was added to the reaction and incubated at RT for 90 min. After incubation, the reaction was immediately cleaned up with a Zymo DNA Clean & Concentrator^TM^-5 column (Zymo Research) and eluted in 15 µL of sterile H_2_O. SybrGreen and loading buffer was added to the DNA and samples were electrophoresed in a 1% agarose TAE gel for 1 hr at 135 V and imaged on a Syngene G:BOX chemiluminescence and fluorescence imager. ImageJ was used to analyze the gel image and the data was graphed using Prism 6 (GraphPad).

### Experimental design & statistical analysis

All quantified values are reported as the mean ± SEM. The relevant sample type and number (N), and statistical tests used to evaluate significance for each experiment are presented with each data set. The sample sizes used in each experiment were based on estimates of expected effect sizes (~10–50%) and standard deviations from preliminary data and were powered to detect differences with a 0.05 significance (α) level and 0.9 power (1-β).

## Supplementary information

Supplemental Figures & Table

## Data Availability

All relevant materials, data and associated protocols will promptly be made available to readers without undue qualifications.
