# Supplementary material for: Alpha-synuclein is a DNA binding protein that modulates DNA repair with implications for Lewy body disorders
Source: Sci Rep. 2019 Jul 29;9:10919. doi: 10.1038/s41598-019-47227-z (PMC6662836; doi:10.1038/s41598-019-47227-z)

Supplementary Data File

Title: Alpha-synuclein is a DNA binding protein that modulates DNA repair with implications for Lewy body disorders

Authors: Allison J. Schaser[+1], Valerie R. Osterberg[+1], Sydney E. Dent[1], Teresa L. Stackhouse[1], Colin M. Wakeham[2], Sydney W. Boutros[3], Leah J. Weston[1], Nichole Owen[4], Tamily A. Weissman[5], Esteban Luna[6], Jacob Raber[3], Kelvin C. Luk[6], Amanda K. McCullough[4,7], Randall L. Woltjer[8], Vivek K. Unni[1,9,*]

Affiliations:

[1]Department of Neurology & Jungers Center for Neurosciences Research; Oregon Health & Science University; Portland, OR, 97239; USA

[2]Neuroscience Graduate Program, Vollum Institute; Oregon Health & Science University; Portland, OR, 97239; USA

[3]Departments of Behavioral Neuroscience, Neurology, and Radiation Medicine and Division of Neuroscience, ONPRC; Oregon Health & Science University; Portland, Oregon, 970239, USA

[4]Department of Molecular and Medical Genetics; Oregon Health & Science University; Portland, OR, 97239; USA

[5]Department of Biology; Lewis & Clark College; Portland, OR, 97219; USA

[6]Department of Pathology and Laboratory Medicine and Center for Neurodegenerative Disease Research; University of Pennsylvania Perelman School of Medicine; Philadelphia, PA, 19104; USA

[7]Oregon Institute of Occupational Health Sciences; Oregon Health & Science University; Portland, OR, 97239; USA

[8]Department of Pathology, Division of Neuropathology; Oregon Health & Science University; Portland, OR, 97239; USA

[9]OHSU Parkinson Center; Oregon Health & Science University; Portland, OR, 97239; USA

[+]These authors contributed equally to this work.

Figure Legends:

Figure 1A Supplemental. Nuclear alpha-synuclein staining is specific with some antibodies but not others in HAP1 cells. Two commercially available alpha-synuclein (αSyn) antibodies, Syn1 and EPR20535, showed clear staining in the nucleus which was essentially abolished in SNCA knock-out (αSyn KO) cells. Three other antibodies, 4B12, 81A and the S129-phospho-synuclein antibody EP1536Y, showed non-specific nuclear staining which did not change appreciably in αSyn KO cells. Scale bar 10μm.

Figure 1B Supplemental. Nuclear alpha-synuclein staining is found in neurons in mouse cortex. Representative image of endogenous alpha-synuclein (αSyn) staining in WT animals showed clear nuclear foci in cortical neurons labeled by the marker NeuN (yellow arrowhead) and much less staining observed in glial cells (NeuN-negative, white arrowhead). Scale bar 10μm.

Figure 5 Supplemental. Alpha-synuclein binds double-stranded DNA. Left: EMSA with 300 bp dsDNA on a 10% polyacrylamide gel shows an increase in shifted species with increasing alpha-synuclein (αSyn) and S129-phospho-synuclein (pSyn) concentration. αSyn causes a larger shift and shifts a higher percentage of DNA than pSyn. Middle: Western blot membrane image of alpha-synuclein antibody staining after transfer of proteins to a nylon membrane. Merged image shows DNA bands (red), αSyn and pSyn protein (green) and colocalization (yellow). Right: Group data showing significant differences between αSyn, pSyn and the control protein glutathione S-transferase (GST, GST gel not shown) at 57 µM: GST fraction shifted = 0.034±0.021, N=3; pSyn = 0.58±0.030, N=3, αSyn =0.86±0.049, N=3, $F(2, 6)=141.4$, $p=0.0001$,

ANOVA, post-hoc Tukey test: GST vs. pSyn p=0.0001, GST vs. αSyn p=0.0001, pSyn vs. αSyn p=0.0034.

<u>Figure 6 Supplemental.</u> **Nuclear alpha-synuclein is rapidly recruited to sites of laser-induced DNA damage in culture.** (A) Mouse cortical neurons imaged in culture expressing S129D, S129A, A53T/S129D, A53T/S129A, or 142E Syn-GFP. Baseline (t=4 sec) and after laser-induced damage (LID, t=6, 12, 18 sec) images show accumulation of the various Syn-GFP forms at DNA damage sites (white arrows). Scale bar 10μm. (B) Left: Group data from different constructs (142E Syn-GFP Enrichment Ratio =2.82±0.32, N=7 cells, S129D Syn-GFP Enrichment Ratio = 4.76±0.83, N=7 cells, S129A Syn-GFP Enrichment Ratio = 2.24±0.53, N=9 cells, A53T/S129D Syn-GFP Enrichment Ratio = 2.53±0.51, N=4 cells, A53T/S129A Syn-GFP Enrichment Ratio = 1.79±0.11, N=4 cells; $F(4,26)=3.865$, p=0.0136, ANOVA, post-hoc Tukey tests: S129D vs. S129A p=0.0177, S129D vs. A53T/S129A p=0.0261, all other comparisons p>0.1298). Right: Group data from S129D, S129A, A53T/S129D, A53T/S129A, and 142E Syn-GFP cultures (S129D Syn-GFP Enrichment Ratio baseline = 1.01±0.03, after LID = 4.76±0.83, N=7 cells, paired t-test p=0.0007; S129A Syn-GFP baseline = 1.05±0.05, after LID = 2.24±0.53, N=9 cells, paired t-test p=0.0399; A53T/S129D Syn-GFP baseline = 0.98±0.05, after LID = 2.53±0.51, N=4 cells, paired t-test p=0.0230; A53T/S129A Syn-GFP baseline = 1.05±0.02, after LID = 1.79±0.11, N=4 cells, paired t-test p=0.0006; 142E Syn-GFP baseline = 1.03±0.03, after LID = 2.82±0.32, N=7 cells, paired t-test p=0.0001).

<u>Figure 7A Supplemental.</u> **Lewy pathology is associated with increased DSBs in mouse hippocampal neurons in culture.** Cultured mouse hippocampal neurons treated with PFFs or PBS show large increases in nuclear γH2AX staining in the PFF-treated group that forms Lewy

pathology 12 days after treatment (PBS = 0.0018 ±0.0004 foci/nucleus, N=3869 cells/4 replicates, PFF = 0.011±.003 foci/nucleus, N=4514 cells/4 replicates, t-test $p<0.0001$). White squares represent areas shown at higher magnification in middle and right images. Scale bar 100μm.

Figure 7B Supplemental. Lewy pathology is associated with increased DSBs in human tissue. Neuronal somatic human Lewy inclusion (pSyn, white arrow) is associated with increased DSBs (γH2AX, yellow arrowhead) compared to cell without Lewy pathology (white arrowhead).

Table 1 Supplemental.

Antibody name	Nuclear labeling WT	Nuclear labeling SNCA KO
**Syn1**	**+**	**-**
**EPR20535**	**+**	**-**
**MJFR1**	**+**	**+**
**EP1536Y**	**+**	**+**

Figure 1A Supplemental

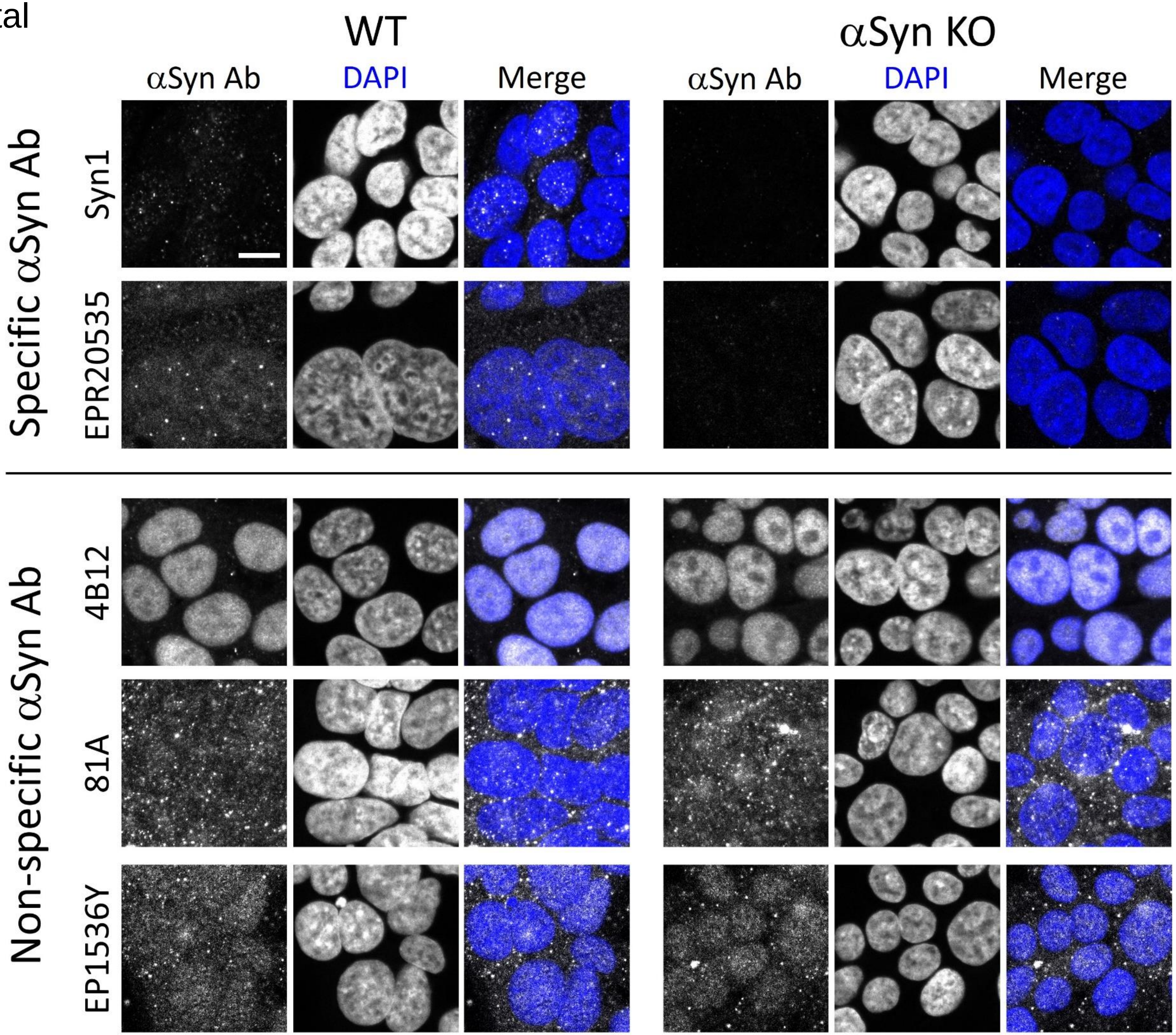

Figure 1B Supplemental

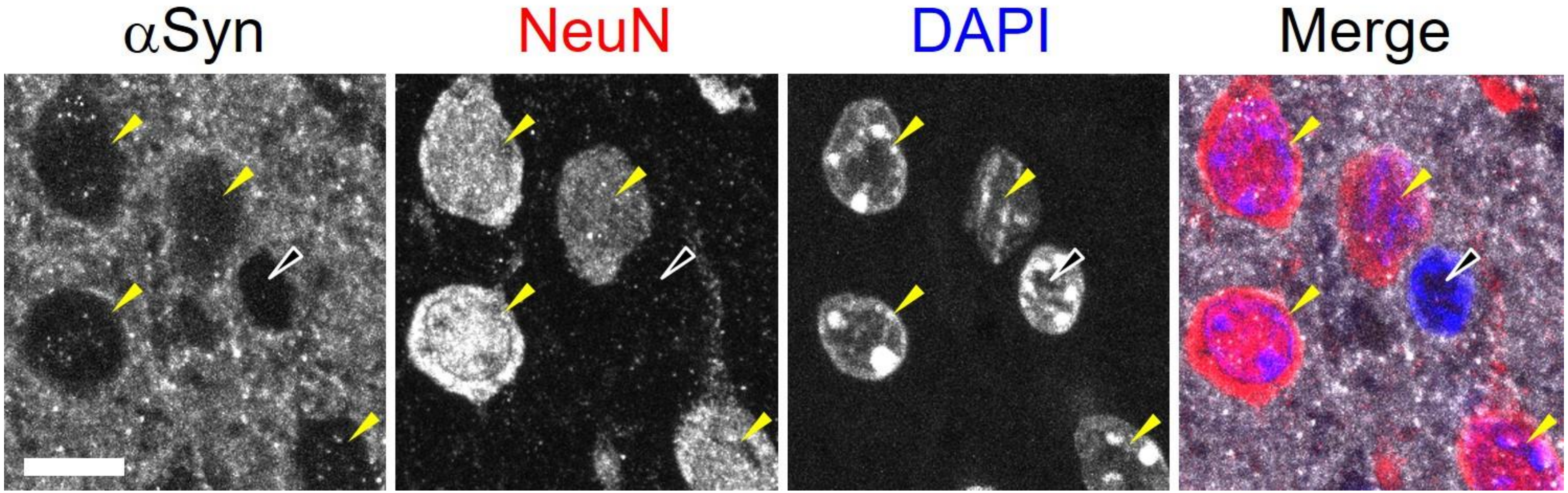

Figure 5 Supplemental

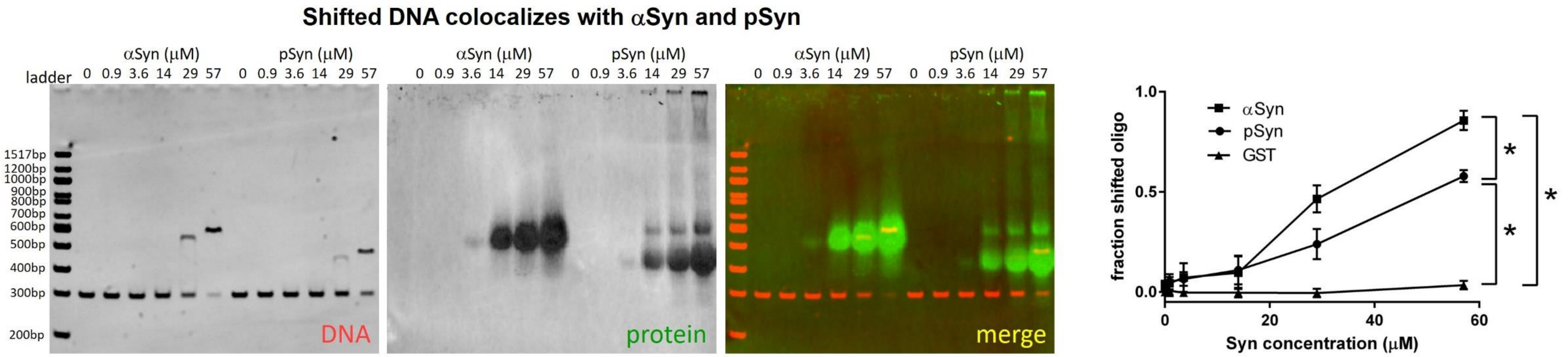

Figure 6 Supplemental

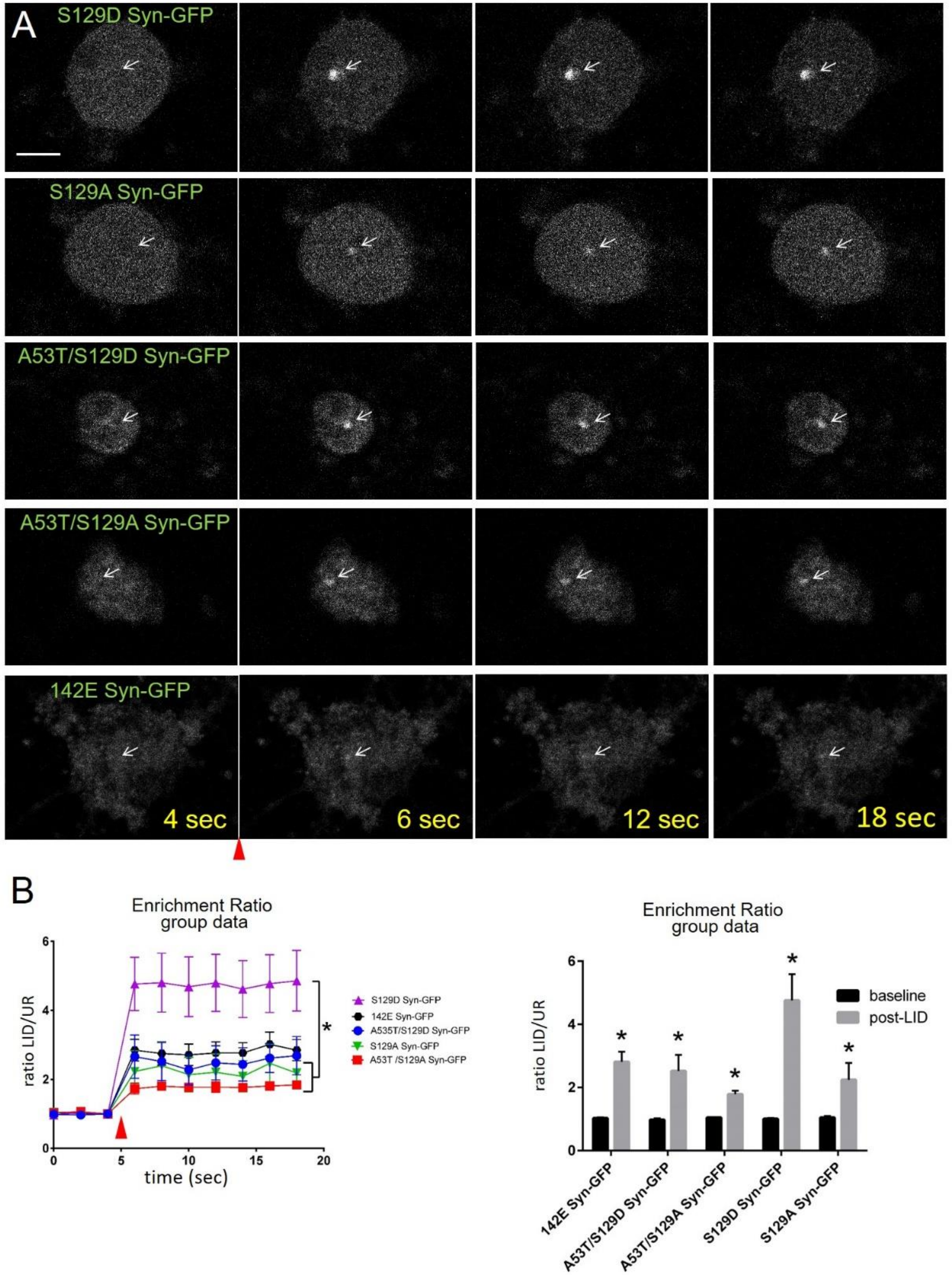

Figure 7A Supplemental

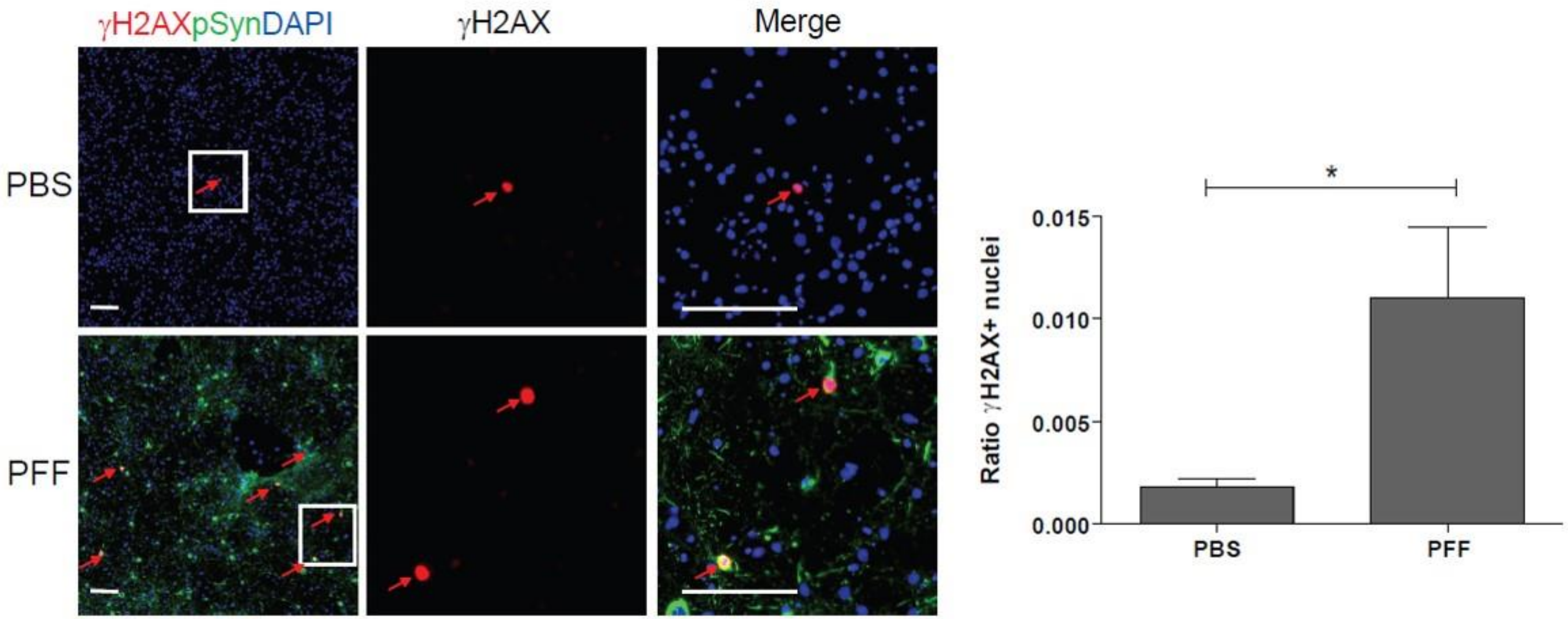

Figure 7B Supplemental

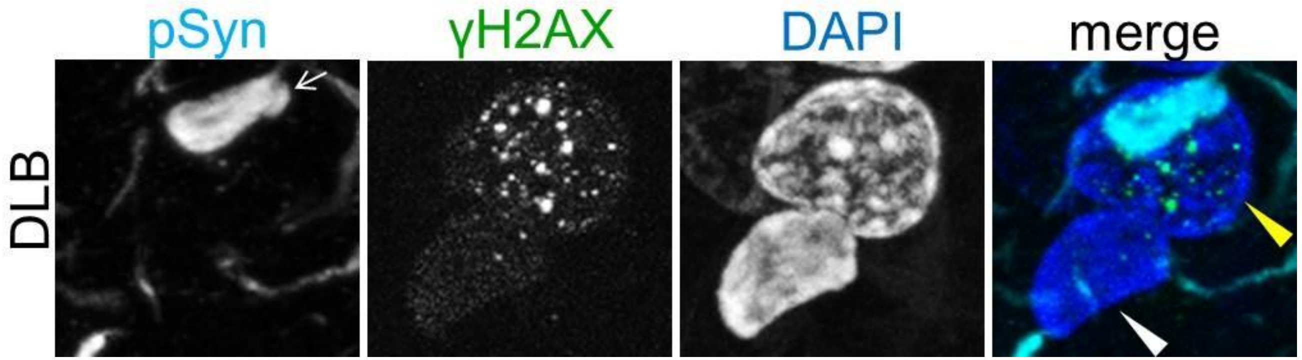

Supplement: Supplementary file 1 — Supplemental Figures & Table [file 41598_2019_47227_MOESM1_ESM.pdf]
